# Sulfated *Cyclocarya Paliurus* Polysaccharide Sorchestrates the Gut Microbiome to Mobilize a Host‐Derived 12‐HEPE Against Ulcerative Colitis

**DOI:** 10.1002/advs.75681

**Published:** 2026-05-15

**Authors:** Xianxiang Chen, Mingyue Shen, Rui Zhang, Zhibing Huang, Hui Niu, Qiang Yu, Yi Chen, Xiangwen Pan, Liyuan Rong, Huiliang Wen, Jun Yang, Jianhua Xie

**Affiliations:** ^1^ State Key Laboratory of Food Science and Resources Nanchang University Nanchang China; ^2^ College of Food Science and Engineering Hainan University Haikou China; ^3^ Department of Clinical Laboratory Medicine Xijing Hospital Fourth Military Medical University Xi'an China; ^4^ Department of Wood Science The University of British Columbia Vancouver Canada

**Keywords:** Cyclocarya paliurus polysaccharide, microbiota, TLR4, UC

## Abstract

Despite extensive evidence supporting the therapeutic potential of natural product‐derived compounds in Ulcerative colitis (UC), their precise mechanisms have yet to be fully elucidated. In this study, structurally modified *Cyclocarya paliurus* polysaccharide (CP) derivatives were evaluated in a dextran sulfate sodium (DSS)‐induced UC mouse model. Among the variants tested, sulfated *Cyclocarya paliurus* polysaccharide (SCP) emerged as the most therapeutically potent. SCP administration markedly attenuated colitis severity, as evidenced by relieved disease symptoms and reinforced intestinal barrier function. Mechanistically, SCP restored gut microbial homeostasis by enriching beneficial *Bacteroidetes* and enhancing short‐chain fatty acids (SCFAs) production. This remodeled microbial ecosystem orchestrates the upregulation of host‐derived 12‐hydroxyeicosapentaenoic acid (12‐HEPE), which exerts anti‐inflammatory effects via direct inhibition of Toll‐like receptor 4 (TLR4) signaling. The gut microbiota's functional relevance was substantiated by fecal microbiota transplantation and antibiotic‐mediated exhaustion studies. Notably, the therapeutic benefits of 12‐HEPE were abrogated upon co‐administration of a TLR4 agonist, confirming its target specificity. Elevated serum 12‐HEPE levels were observed in a human UC cohort, implying a potential compensatory immunoregulatory response. Our findings elucidate a novel microbiota–host interaction axis wherein SCP alleviates UC by modulating the gut microbiota to enhance endogenous 12‐HEPE production, thereby suppressing TLR4‐mediated inflammation.

## Graphical abstract

A sulfated polysaccharide (SCP) from Cyclocarya paliurus alleviates ulcerative colitis by reshaping the gut microbiome. This remodeled microbiota orchestrates the upregulation of the host‐derived lipid 12‐HEPE, which directly inhibits the TLR4/NF‐κB pathway to suppress inflammation. This work reveals a novel microbiota‐host‐lipid axis for treating inflammatory bowel disease.

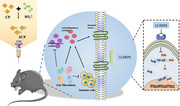

## 1 Introduction

Ulcerative colitis (UC), a recurrent inflammatory disease that affects almost 10 000 000 people worldwide, hallmarked by disabling abdominal pain and immune hyper‐responsiveness [1]. Long‐standing disease carries a step‐wise increase in colorectal cancer risk: 1.6%, 8.3%, and 18.4% after 10, 20, and 30 years, respectively [2]. Current therapeutics achieve remission in only a subset of patients, underscoring the need for mechanism‐based interventions [3]. The gut–immune axis now sits at the center of UC pathogenesis [4]. The abundance of pathogenic bacteria, including *Firmicutes* and other harmful bacteria, is elevated in UC [5]. The gut microbiota as a driving factor is considered in the development of UC [6].

*Cyclocarya paliurus* (Batalin) Iljinskaja (*C. paliurus*) is mainly distributed in southern China and widely utilized in traditional medicine. Its leaves have been historically used as an herbal tea to mitigate elevated blood sugar and inflammation. As a principal active component, *Cyclocarya paliurus* polysaccharide (CP) exhibits antioxidant, anti‐inflammatory, and immunomodulatory properties [7, 8]. However, its poor physico‐chemical properties limit pharmaceutical deployment [9]. Chemical modifications offer a novel and efficient strategy to alter a natural polysaccharide's structure, thereby enabling the tailoring of its properties for broader food applications [9]. Sulfated *Cyclocarya paliurus* polysaccharide (SCP) has a better protective effect on immunosuppressed mice than CP. SCP could also improve body immunity by inhibiting the TLR4/NFκB pathway to reduce intestinal damage [8]. Thus, we hypothesized that SCP ameliorates UC by reconfiguring the gut microbiota–host metabolite–TLR4 nexus.

Dysbiosis is a validated driver of ulcerative colitis (UC). Expansion of pro‐inflammatory *Firmicutes* and contraction of protective *Bacteroidetes* and *Akkermansia*—reflected in an elevated *Firmicutes*/*Bacteroidetes* ratio—correlate with disease activity and promote genotoxic stress via lipopolysaccharide (LPS) and other microbe‐associated molecular patterns [10]. DNA damage in intestinal epithelial cells induced by these microorganisms can be attributed to direct mechanisms, such as LPS production, or indirect mechanisms involving microbial metabolites [11]. Gut microbial metabolites are pivotal in microbiota‐host crosstalk, exerting wide‐ranging effects on host physiology [12].

Microbial metabolites, short‐chain fatty acids (SCFAs), bile acids, and indole derivatives, in inflammatory bowel disease patients are globally depleted in UC; this loss impairs epithelial energy homeostasis, tight‐junction integrity, and eicosanoid balance, thereby fueling chronic inflammation [13]. Compared with those in healthy individuals, SCFA‐producing microorganisms are significantly reduced in patients with an inflammatory bowel and were found to be depleted in the intestinal tract, suggesting their role in UC progression and occurrence [14]. SCFAs normally constrain the eicosanoid cascade; their reduction amplifies pro‐lipid mediators that recruit neutrophils and activate NF‐κB [15, 16]. These metabolite‐driven effects converge on pattern‐recognition receptors, with Toll‐like receptor 4 (TLR4) emerging as the dominant sensor through which dysbiotic microbes propagate colonic inflammation [17].

This research aimed to specify the fine structure of SCP, and used gnotobiotic, antibiotic‐depletion, and fecal‐microbiota‐transplantation models to show that SCP reverses DSS‐colitis exclusively via the microbiota. We demonstrated that SCP mitigated intestinal inflammation by indirectly targeting TLR4. Furthermore, we identified that the gut microbiota remodeled by SCP orchestrates the upregulation of the host‐derived bioactive lipid 12‐HEPE, which was identified as a key mediator conferring SCP's benefits primarily via inhibition of the TLR4‐mediated NFκB/NLRP3 signaling pathway. Our work elucidated a novel pathway of microbiota‐host interaction and positioned SCP as a promising microbiota‐directed therapeutic agent.

## 2 Results

### 2.1 Polysaccharide Structure Determination

To obtain accurate polysaccharide structural information, CP was purified to remove the impurities and eliminate interference using H_2_O_2_ and a DE‐52 column. A highly pure polysaccharide CP1 was obtained. The total sugar of CP1 was 82.14% ± 1.13%. CP1 was mainly composed of Glc (53.57%), Gal (26.70%), Ara (10.23%), Man (9.50%), and the surface was relatively smooth with honeycomb‐like pores. The result of GC‐MS showed that CP1 consisted of T‐Araf (7.36%), T‐Glcp (11.43%), T‐Manp (5.97%), 1,3‐Galp (24.17%), 1,4‐Manp (4.60%), 1,2,5‐Araf (3.70%), 1,6‐Glcp (34.72%), and 1,2,4,6‐Glcp (5.07%) (Table S1). These ratios were similar to the result of monosaccharide composition. The main branching units were 1,6‐Glcp and 1,3‐Galp, which were linked to the O‐2 and O‐4 positions of the 1,6‐Glcp residues.

**FIGURE 1:**
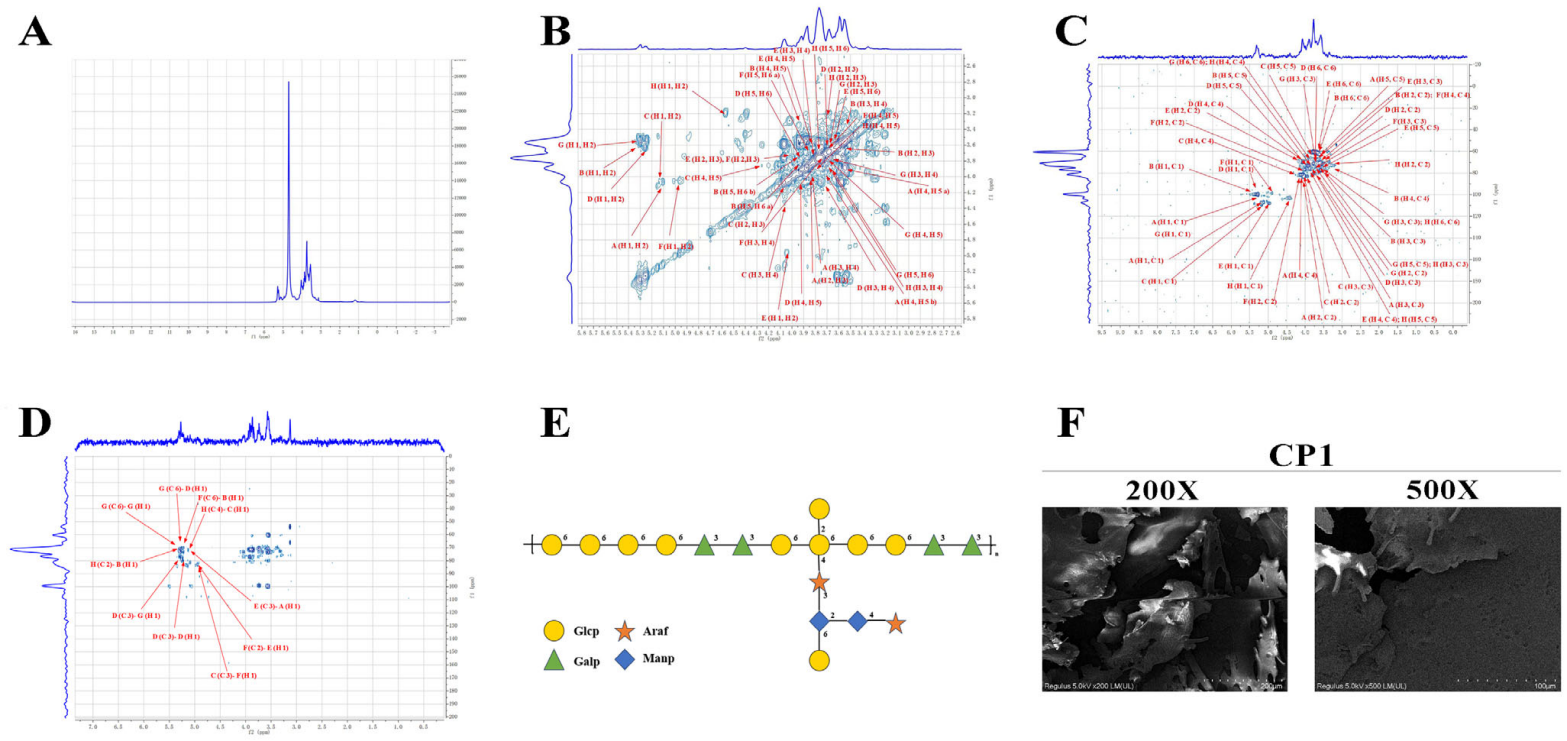
Polysaccharide structure analysis. (A) ^1^H NMR spectrum of CP1; (B) ^1^H‐ ^1^H DQF‐COSY spectrum of CP1; (C) ^1^H‐ ^13^C HSQC correlation spectrum of CP1; (D) The ^1^H‐ ^13^C HMBC spectrum of CP1 recorded at 295 K; (E) Main repeating unit of CP1; (F) Microstructure images of polysaccharide at 200 × and 500 ×.

**FIGURE 2:**
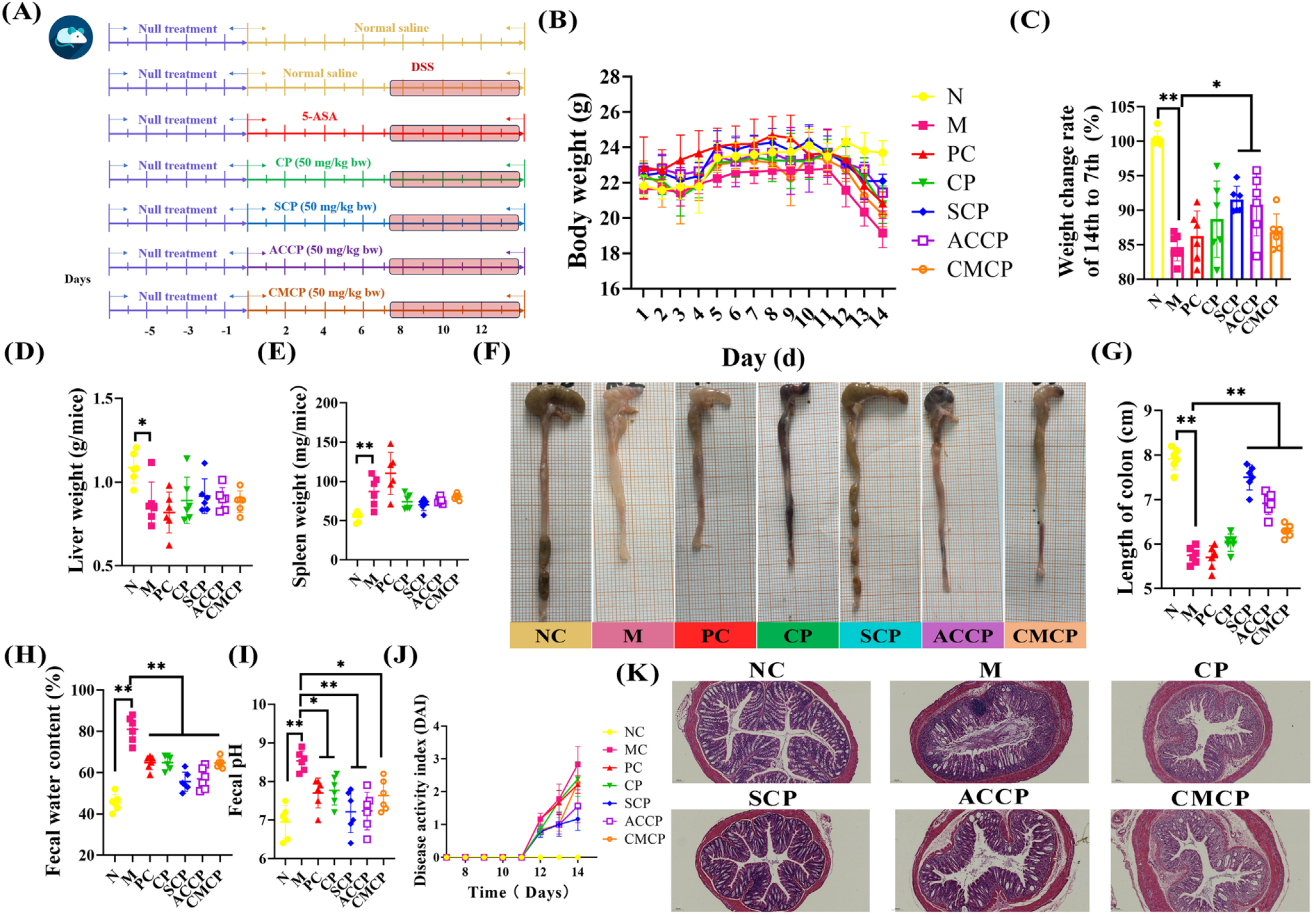
Effects of CP, SCP, ACCP, and CMCP on physiological activities and cytokines in DSS‐induced mice. (A) Schematic of the experimental implementation of polysaccharide on DSS‐treated mice; After 7 days of feeding, all mice were divided into Normal (N) group, Model (M) group, 5‐aminosalicylic acid (5‐ASA) group (PC group), CP group, SCP group, ACCP group, and CMCP group. Saline had been gavaged into N and M groups from the 8th to the 21st. And CP, SCP, ACCP, and CMCP with a concentration of 50 mg/kg bw had been gavaged into the CP group, SCP group, ACCP group, and CMCP group from the 8th to the 21st, respectively. At the same time, the PC group was treated with 5‐ASA for 100 mg/kg·bw. Besides, the 3% DSS was added into drinking water during the last 7 days except N group; (B) Body weight; (C) Body weight change rate of 14th to seventh; (D) Spleen index; (E) Liver index; (F) Representative images of the mouse colon; (G) Colon length; (H,I) Efficacy of polysaccharides on fecal water content, fecal pH values; (J) DAI; The DAI = (body mass index+ fecal consistency score+ anal bleeding score)/3; (K) H&E staining of the colon.

Further, NMR spectroscopy analysis verified the above structural information and indicated the connected pattern of polysaccharide residues. Combined with 1D NMR (Figure 1A) and 2D NMR (Figure 1B–D), eight H1‐H2 coupling resonances were identified. The anomeric protons’ chemical shifts of CP1 residues were assigned (δ 5.11, δ 5.31, δ 4.98, δ 5.26, δ 4.95, δ 5.15, δ 5.33, and δ 4.58 ppm). Furthermore, the other chemical shifts of residue A‐H were assigned by DQF‐COSY and HSQC spectrums [18, 19, 20, 21, 22, 23] and listed in Table S2.

The 2D NMR HMBC spectrum (Figure 1D) can be used to obtain the linkage patterns of CP1. The 1,6‐Glc and 1,3‐Gal alternately linked as the main chain of CP1 were confirmed according to their ratio and the above results. Furthermore, correlations between other residues were assigned. Thus, the fine structure of CP1 was confirmed (Figure 1E). The 1,6‐Glc and 1,3‐Gal in a ratio of 4:2 as the backbone of CP1 was confirmed, and approximately every two circles, the O‐2 and O‐6 position of 1,6‐Glc were substituted. The side chains consist of T‐Araf, T‐Glcp, 1, 4‐Manp, 1, 3‐Araf, and 1, 2, 6‐Manp.

### 2.2 SCP Exhibited Significant Alleviation Against DSS‐Induced Colitis in Mice

The weight loss, loose stool, colon shortening, and pathological changes in the colon are the most remarkable characteristics in colitis [5, 24]. DSS‐induced mice showed grievous pathological signs, including weight loss, liver weight loss, spleen enlargement, shortening of the colon, thinning of stool, enhancing pH of stool, and disease activity index (DAI) scores (Figure 2B–J). However, pretreating polysaccharides could alleviate these changes. Different polysaccharides had different degrees of alleviating effect. The SCP had the best results in those indexes, especially in the index of the length of the colon. The pathological changes of the colon can also be observed visually (Figure 2K). The M group observed severe pathological damage, including inflammatory cell infiltration, villus loss, and crypt destruction. All polysaccharide groups can attenuate these symptoms. Among them, the SCP group had the best protective effect.

RNA‐seq was conducted to explore the molecular mechanisms of polysaccharides in mitigating colitis. In Figure S2A, no evident deflection front was observed in all the groups, indicating that the sequencing was not biased. PCA analysis distinguished clearly all the polysaccharide groups from the M group, reflecting significant differences between them, and the SCP group showed the farthest distance from M compared to the other polysaccharide groups (Figure S2B). The Venn diagram showed similar results (Figure S2C). There were 1762 upregulated, 1011 downregulated genes in the CP group, 3128 upregulated, 2060 downregulated genes in the SCP group, 391 upregulated, 184 downregulated genes in the ACCP group, and 1865 upregulated, 1264 downregulated genes in the CMCP group compared with the M group (Figure S2D). GO analysis of DEGs showed that after pretreatment with the polysaccharides, the main change terms were “cell adhesion”, “regulation of response to stimulus”, “immune response”, and “inflammatory response” (Figure S2F). These results indicated that inflammatory and immune dysregulation played a central role in colitis, and the polysaccharide effectively mitigated these responses. KEGG analysis found that MAPK, NF‐κB, and ECM pathways were significantly enriched (Figure S2G,H).

T cells and macrophages were stained and counted to verify the immunoregulation effect and changes in goblet cells. The goblet cells were significantly decreased, and CD4^+^, CD8^+,^ and macrophages were significantly increased after DSS treatment. However, all polysaccharide groups could reverse these trends of goblet cells, CD4^+^ cells, CD8^+^ cells, and macrophages (Figure S3A–E). For inflammatory cytokines, in the M group, the TNF‐α and IL‐1β levels were enhanced, while the polysaccharides administration groups reduced these levels. In particular, SCP exhibited significant reductions of TNF‐α (*p* < *0.05*) and IL‐1β (*p* < *0.01*) levels (Figure S3F,G). Moreover, a similar trend was found in MPO, SIgA, and TGF‐β3 (Figure S3L–N). As for anti‐inflammatory cytokines (IL‐4, IL‐13, IL‐17, and IL‐22), these levels were reduced in the M group, while SCP administration groups could downregulate them (IL‐4 (*p* < *0.05*), IL‐13, IL‐17 (*p* < *0.05*), and IL‐22) (Figure S3H–K). In addition, a similar trend was found in DAO level (Figure S3O). In Figure S3P,Q, the levels of LBP and LPS were increased in colitis mice. However, the treatment diminished this promotion. The tight junctional proteins zonulin, occludin‐1 (ZO‐1), Occludin, and Claudin‐1 were significantly reduced in the M group, compared with the N group. whereas this reduction effect was diminished in the polysaccharides treatment group, especially in the SCP group (Figure S3R–U). IHC result for MUC‐2 protein verified the above results for intestinal barrier function (Figure S3V,W). Damage to the intestinal barrier function will result in harmful substances entering the bloodstream, which in turn will lead to systemic inflammation. The T‐AOC, CAT, and SOD expression in the liver was significantly decreased, and the MDA pruduction was significantly increased in colitis mice, compared with the N group. However, SCP groups can reverse these trends (Table S4).

To further explore the mechanism of *C. paliurus* polysaccharide on colitis mice and verify the result of KEGG, Western blot was used to measure the protein levels on MAPK, NF‐κB, and ECM pathways. DSS increased the phosphorylation levels of Jnk, Erk, P38, SMAD2, SMAD3, and NF‐κB. Moreover, the expressions of TGF‐β, SMAD4, and MyD88 under the influence of DSS were increased. However, the administration of CP, SCP, ACCP, and CMCP can reverse this trend. These results indicated that CP, SCP, ACCP, and CMCP alleviated colitis in mice through similar pathways (Figure S4A–K). This commonality is likely attributable to their shared origin and similar chemical structures.

SCFAs (acetic acid, propionic acid, isobutyric acid, butyric acid, isovaleric acid, and valeric acid) are produced by polysaccharides fermented in gut microbiota [25]. The SCFAs in colitis mice were significantly (*p* < *0.01*) reduced in the cecum compared with the N group. Nevertheless, considerably enhanced levels of SCFAs were found in all the polysaccharide groups (Table S5).

**FIGURE 3:**
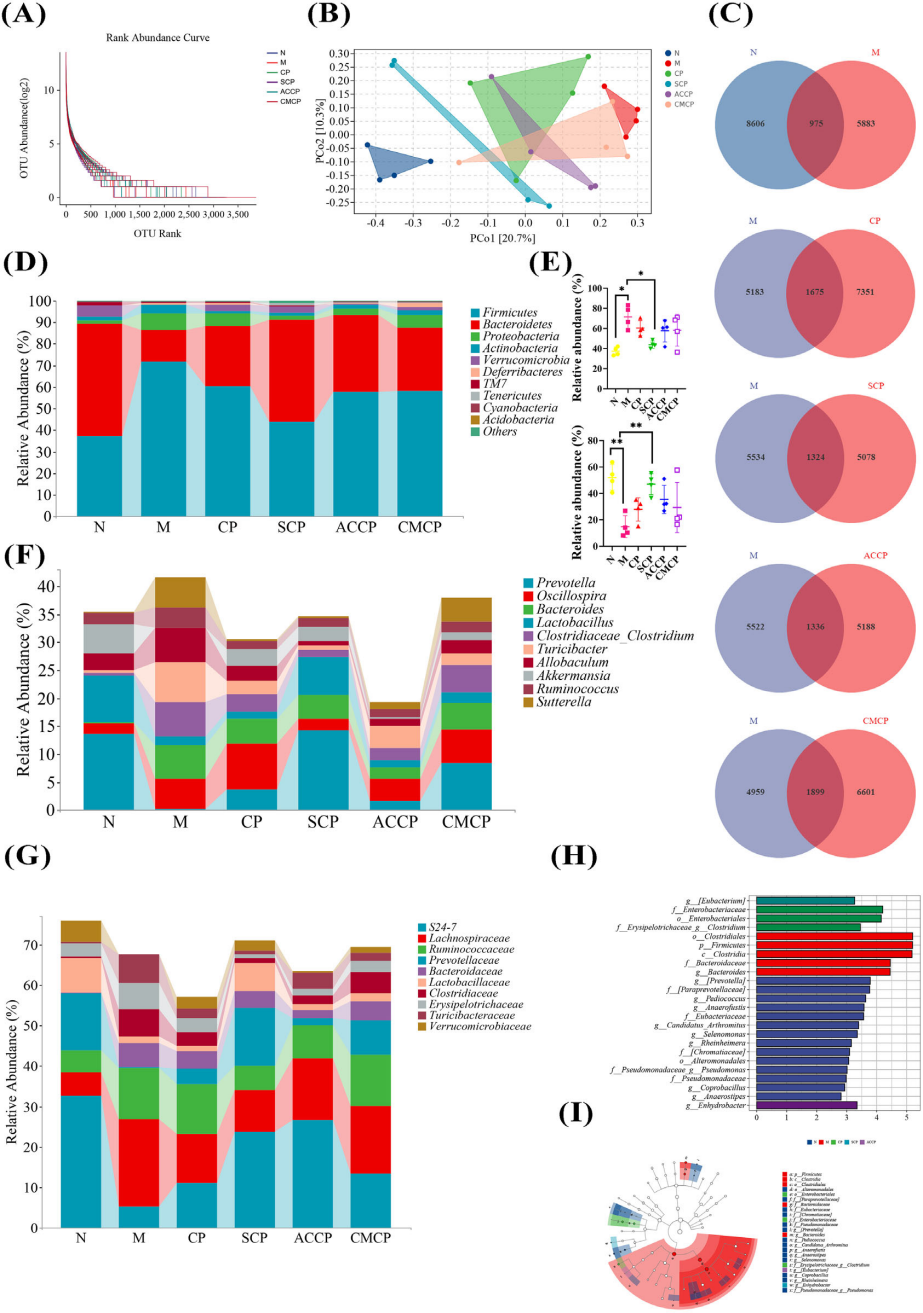
Efficacy of CP, SCP, ACCP, and CMCP on gut microbiota diversities and compositions. (A) Rank‐abundance curve; (B) PCoA analysis based on Bray–Curtis distance, the confidence interval is 95%; (C) Venn diagram; Each block represents a group, the overlapping area between the blocks indicates the ASV/OTU shared between the corresponding groups, and the number of each block indicates the number of ASV/OTU contained in that block; (D) Relative abundances of the gut microbiota at the phylum level. Change in relative abundances of the gut microbiota at the phylum level in each group. Data are the means ± s.e.m. each color represents one bacterial phylum; (E) Representative gut microbiota change at the phylum level. (F) Relative abundances of the gut microbiota at the family level. Change in relative abundances of the gut microbiota at the family level in each group; Data are the means ± s.e.m. each color represents one bacterial family; (G) Relative abundances of the gut microbiota at the genus level. Change in relative abundances of the gut microbiota at the genus level in each group; Data are the means ± s.e.m. each color represents one bacterial genus; (H) Dendrogram of gut microbiota classification. (I) Taxonomic cladograms were generated by LefSe of metagenomic analysis data; The size of each circle is proportional to the taxon's abundance.

The gut microbiota of colitis mice pretreated with polysaccharides was assessed. The flattened waves in the rank abundance curves indicated that these samples were homogeneous and abundant in species compositions (Figure 3A). Principal coordinate analysis (PCoA) showed that there is some distance between the N group and the M groups, indicating that the gut microbiota had changed when treated with DSS. After feeding polysaccharides, all groups moved closer to the N group, with the SCP group being closest (Figure 3B). The Venn diagram showed the common and unique OTUs between each group (Figure 3C). In Figure 3D,E, the relative abundance of *Firmicutes* and *Bacteroidetes* accounted for up to 80% to the total microorganisms, making them the most common species found in the colitis model. After drinking with DSS, a significant increase in the abundance of *Firmicutes* (*p* < *0.05*), concurrently with a marked decrease in *Bacteroidetes* (*p* < *0.01*) relative to the N group. These trends were significantly reversed upon pretreatment with SCP (*p* < *0.05*). At the phylum level, the SCP group showed the most similarity to the N group. Figure 3F,G exhibited the components of gut microbiota at the genus and family levels. After DSS treatment, the microorganisms had changed. However, when the mice were pretreated with polysaccharides, this phenomenon was mitigated. Among them, the SCP group showed the greatest similarity to the N group in terms of components or ratio. The different compositions of gut microbiota among all groups were identified by Linear discriminant analysis effect size (LEfSe) analysis (Figure 3H,I). *g_Prevotella* and *f_Eubacteriaceae* were dominant in the N group. *p_Firmicutes* and *c_Clostridiales* were dominant in the M group. *g_Eubacterium* was dominant in the SCP group. Overall, these results demonstrated that SCP exhibited the best alleviating effect on colitis mice, and the gut microbiota may play a key role in this process.

### 2.3 Sulfate Substitution Site Confirmation

Furthermore, sulfated modification was carried out on CP1, and SCP1 was obtained. The appearance of two distinct peaks at 1249 cm^−^^1^ (S─O asymmetric stretching) and 819 cm^−^^1^ (C─O─S symmetric stretching) in SCP1, which were absent in CP1, confirmed the successful sulfation modification of the polysaccharide (Figure S1A). The total sugar of SCP1 was 80.38% ± 1.11%. The CP1 and SCP1 had similar composition. SCP1 was mainly composed of Glc (53.99%), Gal (28.14%), Ara (10.22%), and Man (8.12%) (Table S1). In addition, SCP1 was relatively smooth surface, which was similar to CP1. These indicated that t CP1 only changes the group, and its structure was unchanged in the modified process. The 2D NMR HSQC spectrum showed that at 84.71, 83.82, and 80.11 ppm (Figure S1B), these peaks were weakened obviously, which indicated that C4 in residue A, C2 in residue A, and C2 in residue C were primarily substituted by the sulfate group (Figure S1C), respectively. In addition, the apparent morphology of SCP was similar to that of CP, indicating that less effect on the apparent structure of CP was the change in the modified process (Figure S1D).

### 2.4 The Alleviated Effect of SCP was Correlated With Gut Microbiota

**FIGURE 4:**
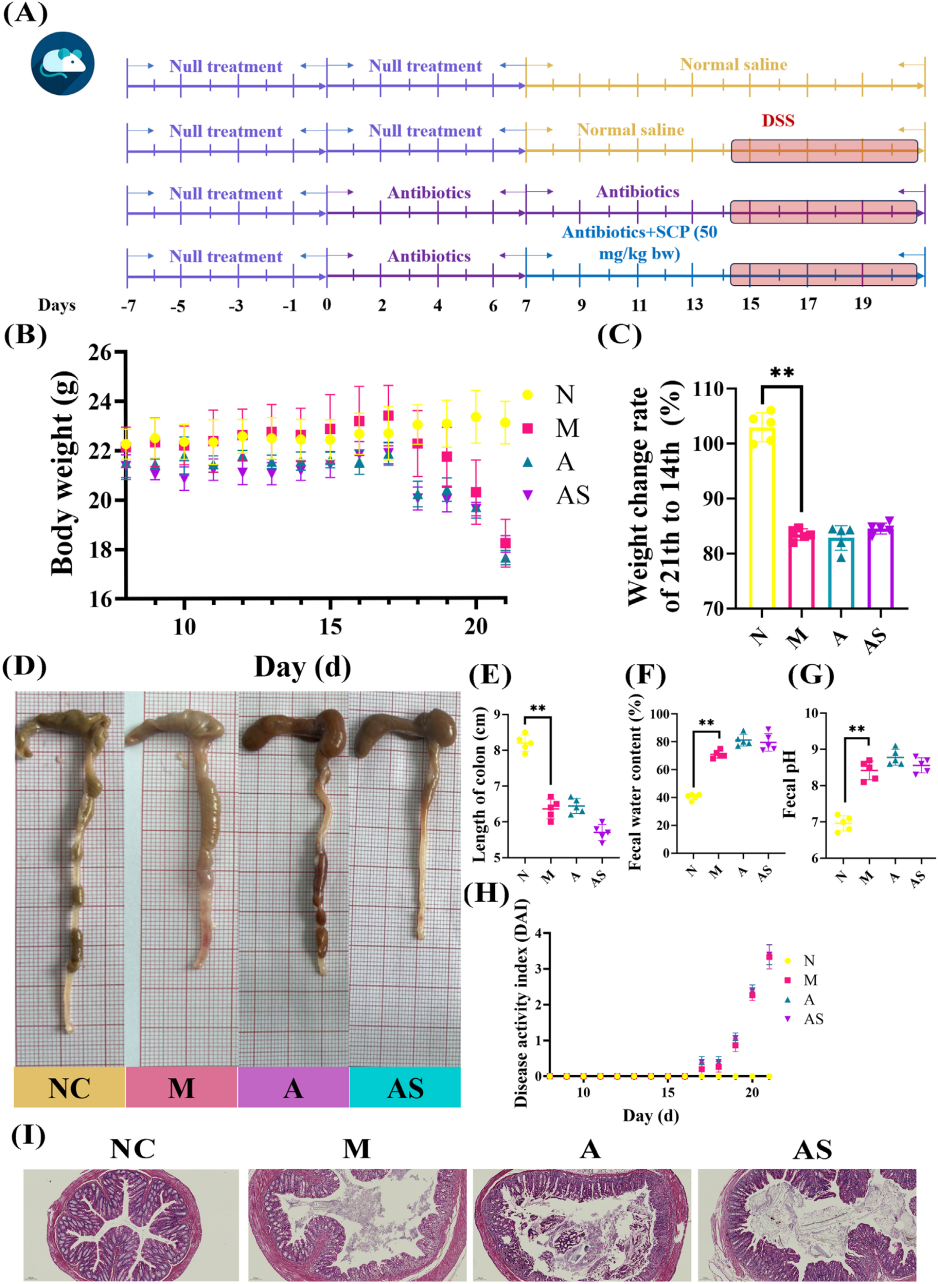
The alleviated effect of SCP was correlated with gut microbiota in the antibiotic cocktail experiment. (A) Schematic of the experimental implementation of polysaccharide on DSS‐treated mice; Mice were randomly divided into four groups: Normal (N) group, Model (M) group, Antibiotics (A) group, and Antibiotics + SCP (AS) group. Saline had been gavaged into N and M groups from 8th to 21st. A group and an AS group were given 0.2 mL of antibiotics and 0.1 mL of antibiotics + 0.1 mL of 50 mg/kg SCP solution, respectively. Besides, the 3% DSS was added into drinking water during the last 7 days except N group; (B) Body weight; (C) Body weight change rate of 21st–14th; (D) Representative images of the mouse colon; (E) Colon length; (F‐G) Efficacy of SCP on fecal water content, fecal pH values; (H) DAI; The DAI = (body mass index+ fecal consistency score+ anal bleeding score)/3; (I) H&E staining of the colon.

The gut microbiota is susceptible to antibiotics. Thus, gut microbiota was removed by antibiotic cocktail therapy [26]. To confirm if the gut microbiota is the key point in treating colitis by SCP, the antibiotic cocktail was administered by gavage to the mice with DSS‐induced colitis. As colitis progressed, the mice's weight of M group, A group and AS group was reduced quickly. However, the decrease in mouse weight was much faster in colitis mice when they were treated with an antibiotic cocktail (Figure 4B,C). In Figure 4D,E, the colon length did not have an obvious change between antibiotic cocktail‐treated colitis mice and colitis mice. However, we found that with or without gavage of polysaccharides, there was a very large accumulation of blood in the cecum of antibiotic‐treated mice. Both fecal water and fecal pH changes were increased in antibiotic cocktail‐treated mice compared with colitis (Figure 4F,G). The DAI result was similar to the above results (Figure 4H). H&E staining analysis demonstrated inflammatory infiltration and disruption of intestinal structure in colitis mice. Antibiotic‐treated mice exacerbated these phenomena. However, SCP did not alleviate these symptoms in antibiotic‐treated colitis mice.

To verify whether removing gut microbiota whether influence immunoregulation effect, the change in goblet cells and T cells was stained and calculated. The goblet cells were significantly decreased, and CD4^+^ and CD8^+^ were increased after drinking water with DSS. However, the decrease in goblet cells and increase in CD4^+^ and CD8^+^ cells were much more severe in colitis mice treated with an antibiotic cocktail, and SCP had no alleviating effect on those parameters (Figure S5A–D). As for inflammatory cytokines (TNF‐α, IL‐1β, and IFN‐γ), these levels were significantly enhanced (*p* < *0.05*) in the M group. However, these inflammatory cytokines were further produced when treated with an antibiotic cocktail in colitis mice, especially IFN‐γ(*p* < *0.05*) and TNF‐α (*p* < *0.05*). In addition, SCP did not slow this growth (Figure S5E–G). Moreover, a similar trend in the level of MPO (Figure S5M), SIgA (Figure S5N), LPS (Figure S5P), LBP (Figure S5Q) and CCL2 (Figure S5R) was found, especially MPO (*p* < *0.05*), LPS (*p* < *0.05*), LBP (*p* < *0.05*), and CCL2 (*p* < *0.05*). As for anti‐inflammatory cytokines (IL‐2, IL‐4, IL‐6, IL‐13, and IL‐22), these levels were reduced in the M group, while this reduction was further exacerbated when treated with an antibiotic cocktail in colitis mice. Moreover, a similar trend in DAO was found. Moreover, the treatment of SCP did not achieve any effect when the mice had their gut microbiota removed. The intestinal barrier function analysis found that the tight junctional proteins Occludin and Claudin‐1 were significantly reduced in colitis mice, compared with the N group. However, the functional intestinal barrier was more severely disrupted in the A group and the AS group (Figure S5S–U). The results determined by IHC in MUC‐2 protein were verified by the above results of intestinal barrier function (Figure S5V,W).

Western blot results showed that the NFκB and ECM pathways were further activated by the antibiotic cocktail in colitis mice (Figure S6A–J). SCFAs are produced by polysaccharide fermented in the gut microbiota. Thus, measurement of SCFAs can indirectly demonstrate whether the gut microbiota has been removed to verify the accuracy of the above results. In Table S6, the acetic acid, propionic acid, isobutyric acid, butyric acid, isovaleric acid, and valeric acid in the M group were significantly (*p* < *0.01*) reduced in the cecum compared with the N group. With the addition of an antibiotic cocktail, SCFAs continued to decline to half of what they were in the M group.

### 2.5 Fecal Microbiota Transplantation Mimicked the Alleviating Effect of SCP Treatment

**FIGURE 5:**
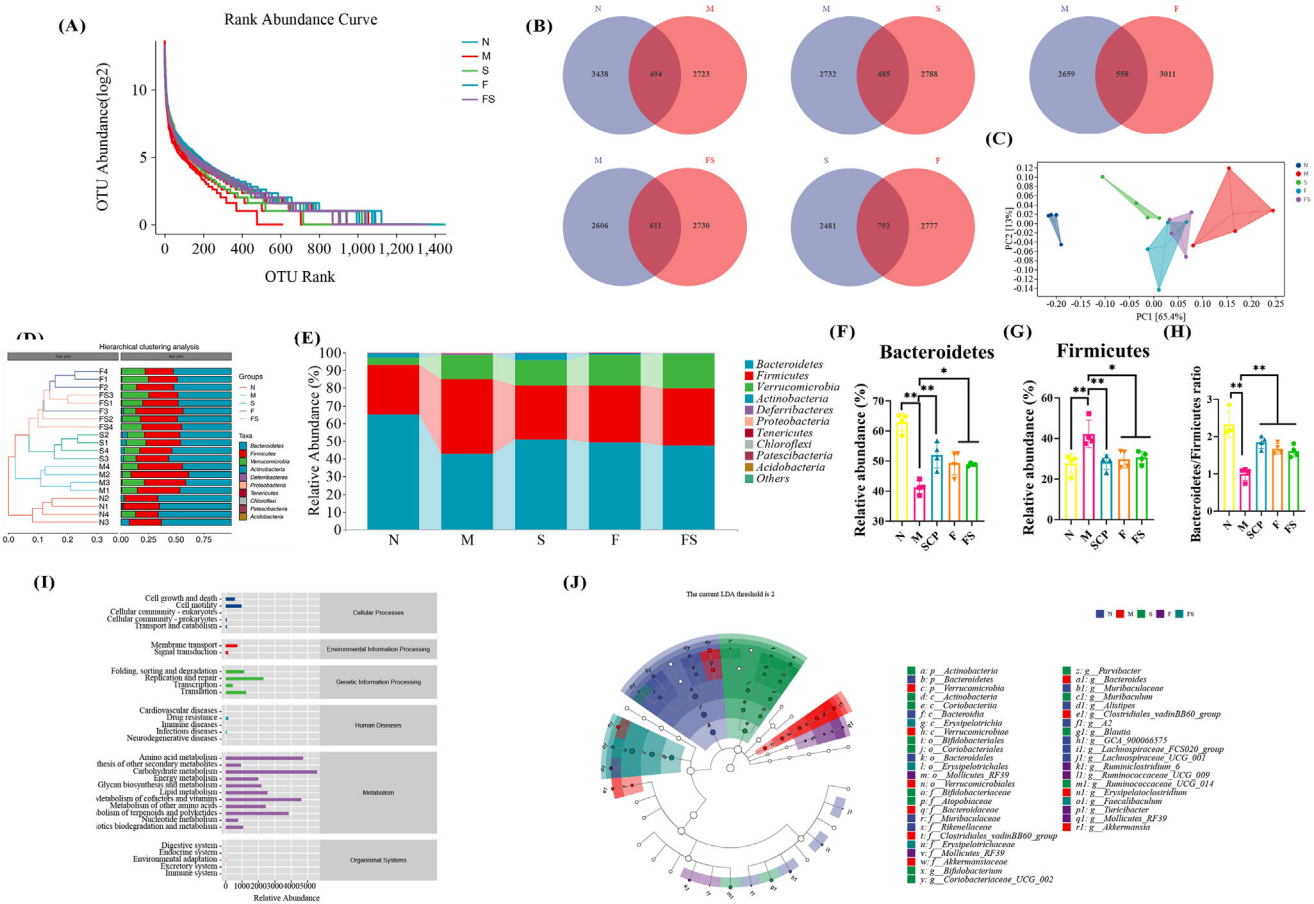
Efficacy of fecal microbiota transplantation on gut microbiota diversities and compositions. (A) Rank‐abundance curve; (B) Venn diagram; Each block represents a group, the overlapping area between the blocks indicates the ASV/OTU shared between the corresponding groups, and the number of each block indicates the number of ASV/OTU contained in that block; (C) PCoA analysis based on Bray–Curtis distance, the confidence interval is 95%; (D) Hierarchical cluster assay; (E) Relative abundances of the gut microbiota at the phylum level. Change in relative abundances of the gut microbiota at the phylum level in each group. Data are the means ± s.e.m. each color represents one bacterial phylum; (F–H) Representative gut microbiota change at the phylum level; (I) PICRUSt prediction of microbial function by KEGG analysis; Castor hidden state prediction algorithm was used to predict the closest sequence species of the feature sequence according to the copy number of the gene family corresponding to the reference sequence in the evolutionary tree, and then the copy number of its gene family was obtained. The gene families are “mapped” to various databases, using MinPath to infer the presence of metabolic pathways; (J) Taxonomic cladograms were generated by LefSe of metagenomic analysis data; The size of each circle is proportional to the taxon's abundance.

**FIGURE 6:**
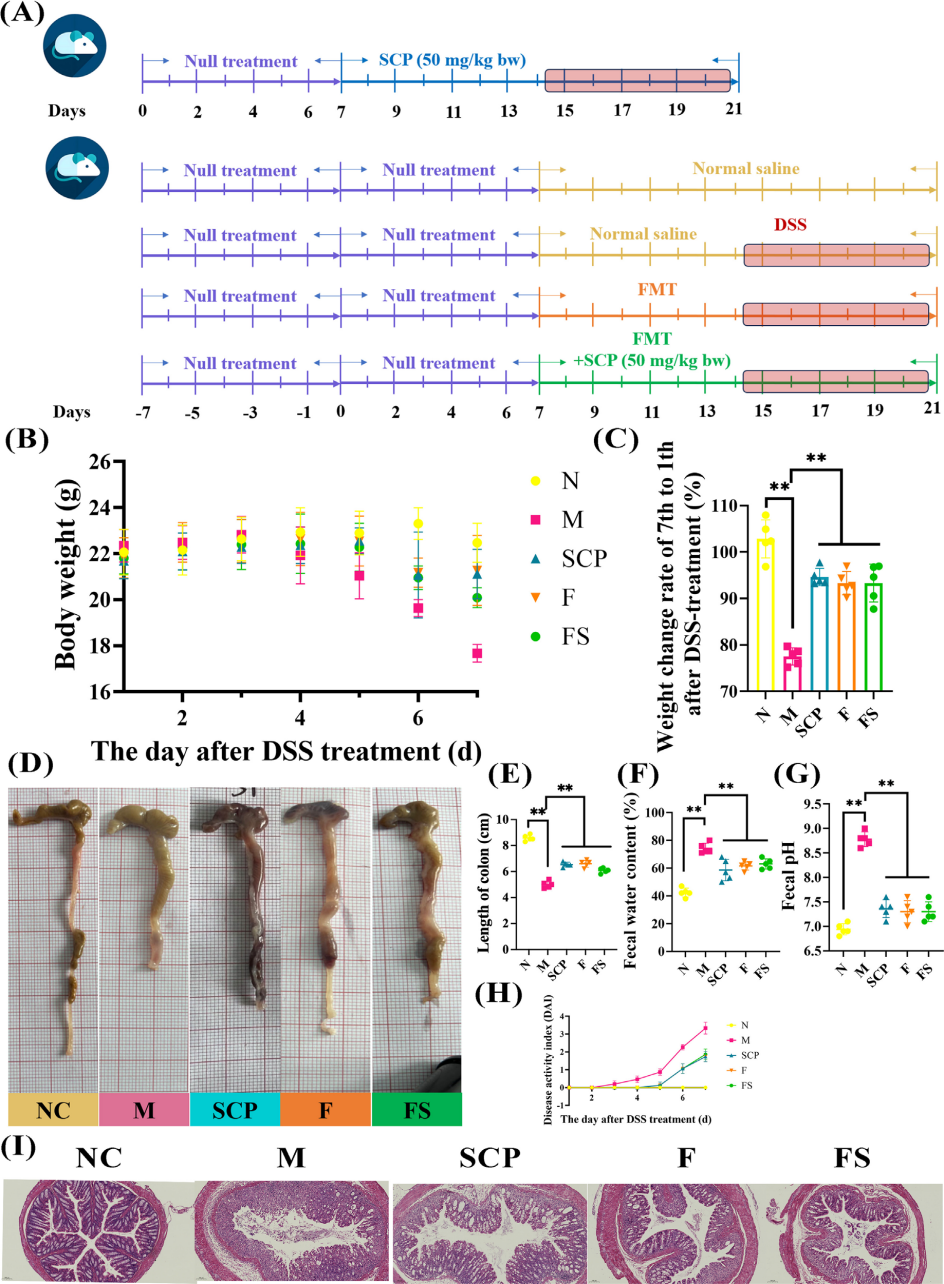
The alleviated effect of SCP was correlated with gut microbiota by the fecal microbiota transplantation experiment. (A) Schematic of the experimental implementation of polysaccharides on DSS‐treated mice. In the study of fecal microbiome transplantation, mice were randomly divided into five groups: Normal (N) group, Model (M) group, SCP group, FMT group (fecal microbiome transplantation of SCP mice before DSS treatment), and FMT + SMP group. Saline had been gavaged into N and M groups from the 8th to the 21st. The FMT group and the FMT + SMP group were given 0.2 mL fecal solution and 0.1 mL fecal solution + 0.1 mL 50 mg/kg SCP solution, respectively. Besides, the 3% DSS was added into drinking water during the last 7 days except N group; (B) Body weight; (C) Body weight change rate of seventh to first after DSS treatment; (D) Representative images of the mouse colon; (E) Colon length; (F‐G) Efficacy of SCP and SCP fecal microbiota transplantation on fecal water content, fecal pH values; (H) DAI; The DAI = (body mass index+ fecal consistency score+ anal bleeding score)/3; (I) H&E staining of the colon.

To verify that gut microbiota mediates the alleviating effect of SCP and SCP as a prebiotic in this process, the DSS‐induced colitis mice were pretreated with the fecal microbiota from SCP. 16S rRNA sequencing was used to determine whether the fecal microbiota transplantation was successful. In Figure 5A, the flattened waves found in the rank abundance curves indicated that samples were homogeneous in species compositions. As expected, the microbiological compositions between the SCP group and the F group were similar at the genus level, especially for *Firmicutes* and *Bacteroidetes* in fecal microbiota transplantation mice, as the two most abundant types of bacteria had the same trend with the SCP group (Figure 5B–E). These results indicated that the fecal microbiota transplantation was successful. Moreover, the result of SCFAs also verified that fecal microbiota transplantation is successful. Compared with the M group, the SCFAs in the F and FS groups were significantly (*p* < *0.01*) recovered in the cecum (Table S7). In addition, fecal microbiota transplantation exhibited a similar alleviating effect on body weight, length of colon, fecal water content, fecal pH, and DAI index compared with the SCP group. Although the FS group also exhibited significant alleviation in these indexes compared with the M group, it did not have an obvious difference from the F group (Figure 6B–H). This may indicate that SCP only acts as a prebiotic in this process and does not relieve colitis by other means. Disorganized glandular arrangement, inflammatory infiltration, and disruption of intestinal structure were recovered, proving that fecal microbiota transplantation could mimic the alleviated effect similar to SCP treatment (Figure 6I). Immunoregulatory effects were measured by observing the changes in goblet cells and T cells, which also verified that fecal microbiota transplantation can enhance the number of goblet cells and reduce CD4^+^ and CD8^+^ cells (Figure S7A–D). Compared with the M group, the expression of TNF‐α (Figure S7E), IL‐1β (Figure S7F), IFN‐γ (Figure S7G), MPO (Figure S7M), SIgA (Figure S7N), LPS (Figure S7P), LBP (Figure S7Q), and CCL2 (Figure S7R) in SCP‐trans mice was significantly reduced. The levels of IL‐2 (Figure S7H), IL‐4 (Figure S7I), IL‐6 (Figure S7J), IL‐13 (Figure S7K), IL‐22 (Figure S7L), and DAO (Figure s7 O) in F and FS groups were improved compared with DSS‐induced colitis mice, with a similar level to the SCP group. These indicated that the transfer could inhibit inflammasome activation. For intestinal barrier functions, the Occludin and Claudin‐1 expression were significantly recovered (*p* < *0.01*) in fecal microbiota transplantation mice compared with the DSS‐induced colitis mice (Figure S8A–C). The results determined by IHC in MUC‐2 protein were verified by the above results of intestinal barrier function (Figure S8D,E). To determine whether the alleviated mechanism of fecal microbiota transplantation in colitis mice was the same as the SCP‐treatment, the related protein of NF‐κB and ECM pathways was measured by Western blot. Compared with the M group, fecal transplantation inhibits the phosphorylation of NF‐κB, iκBα, SMAD2, and SMAD3 and expression of TLR4, Myd88, TGF‐β3, and SMAD4 in colitis mice, just like SCP‐treatment (Figure S8A,F–N).

### 2.6 Gut Microbiota Remodeling by SCP Promotes Host Production of 12‐HEPE

Previous untargeted metabolomics research found that eicosanoid‐related metabolites predominate in the development of colitis [27, 28]. To evaluate the influence of gut microbiota in colitis progression, targeted UPLC‐MS metabolomics was performed using serum and liver samples of mice. Interestingly, the important metabolic 12‐HEPE was enriched in SCP‐treated mice both in the liver and serum. Furthermore, its intensity was increased in fecal microbiota transplantation mice. Amazingly, 12‐HEPE was significantly reduced in the A group and colitis mice both in the liver and serum (Figure S9A–E). Taken together, 12‐HEPE may be a significant host‐derived metabolite mediated by gut microbiota and responsible for the protective effect of SCP in colitis. In addition, the correlations between the gut microbiota and 12‐HEPE were analyzed using Spearman's correlation analysis. *Bacteroidetes* and *Actinobacteria showed* positive correlations with 12‐HEPE, and only *Bacteroidetes* showed significantly positive correlations with 12‐HEPE both in liver and serum (Figure S9F). These results indicated that *Bacteroidetes* may be the key bacteria for the host to produce the 12‐HEPE.

### 2.7 Supplement of 12‐HEPE Could Alleviate DSS‐Induced Colitis

**FIGURE 7:**
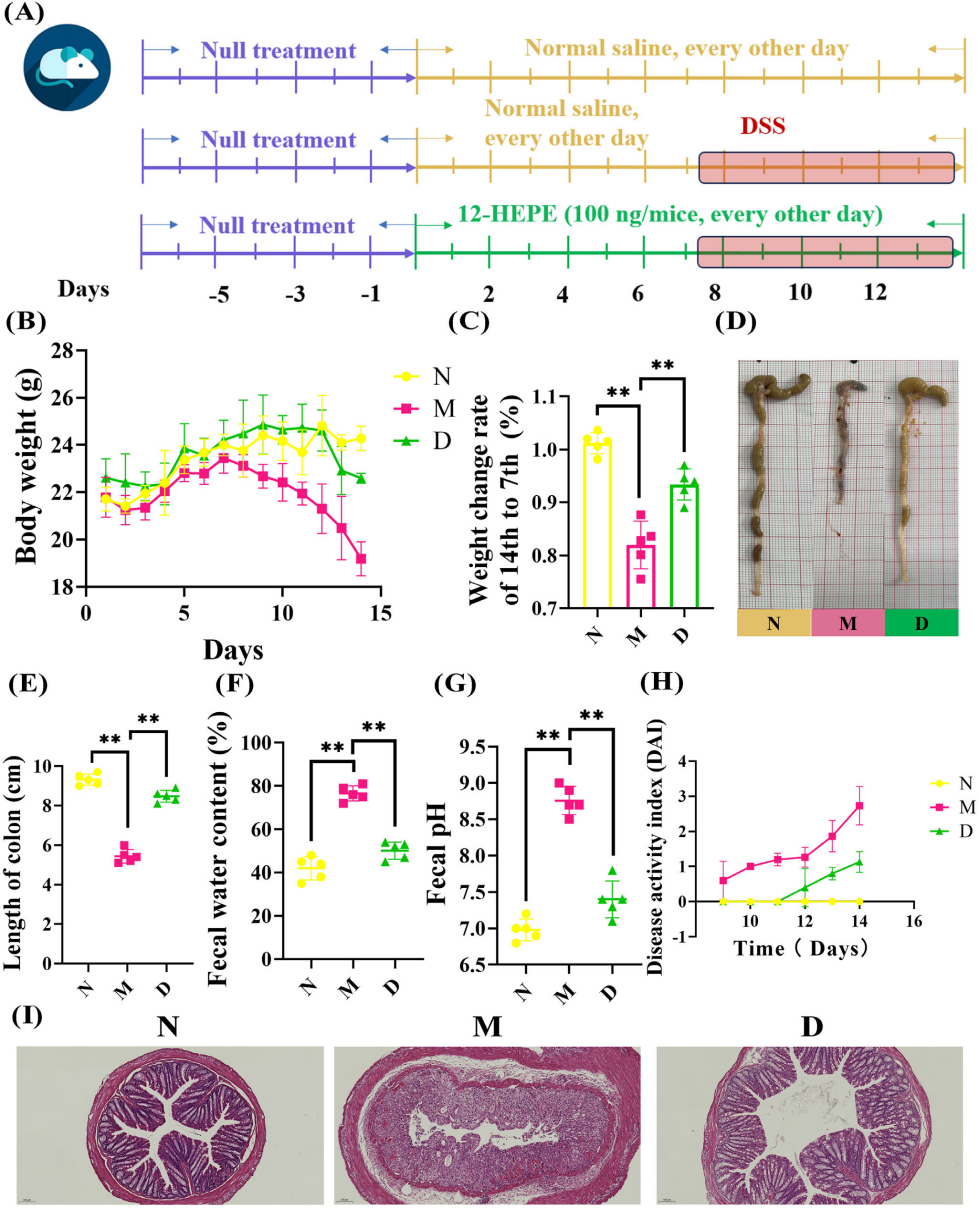
The alleviated effect of 12‐HEPE on colitis. (A) Schematic of the experimental implementation of polysaccharide on DSS‐treated mice; In the study of metabolite injection experiment, mice were randomly divided into three groups: Normal (N) group, Model (M) group, 12‐HEPE metabolite injection (D) group. Saline had been injected into N and M groups every other day from the 8th to the 21st. And 0.2 mL 12‐HEPE at a concentration of 100 ng/mL had been injected into the D group. Besides, the 3% DSS was added into drinking water during the last 7 days except N group; (B) Body weight; (C) Body weight change rate of 14th to seventh^;^ (D) Representative images of the mouse colon; (E) Colon length; (F‐G) Efficacy of 12‐HEPE on fecal water content, fecal pH values; (H) DAI; The DAI = (body mass index+ fecal consistency score+ anal bleeding score)/3; (I) H&E staining of the colon.

**FIGURE 8:**
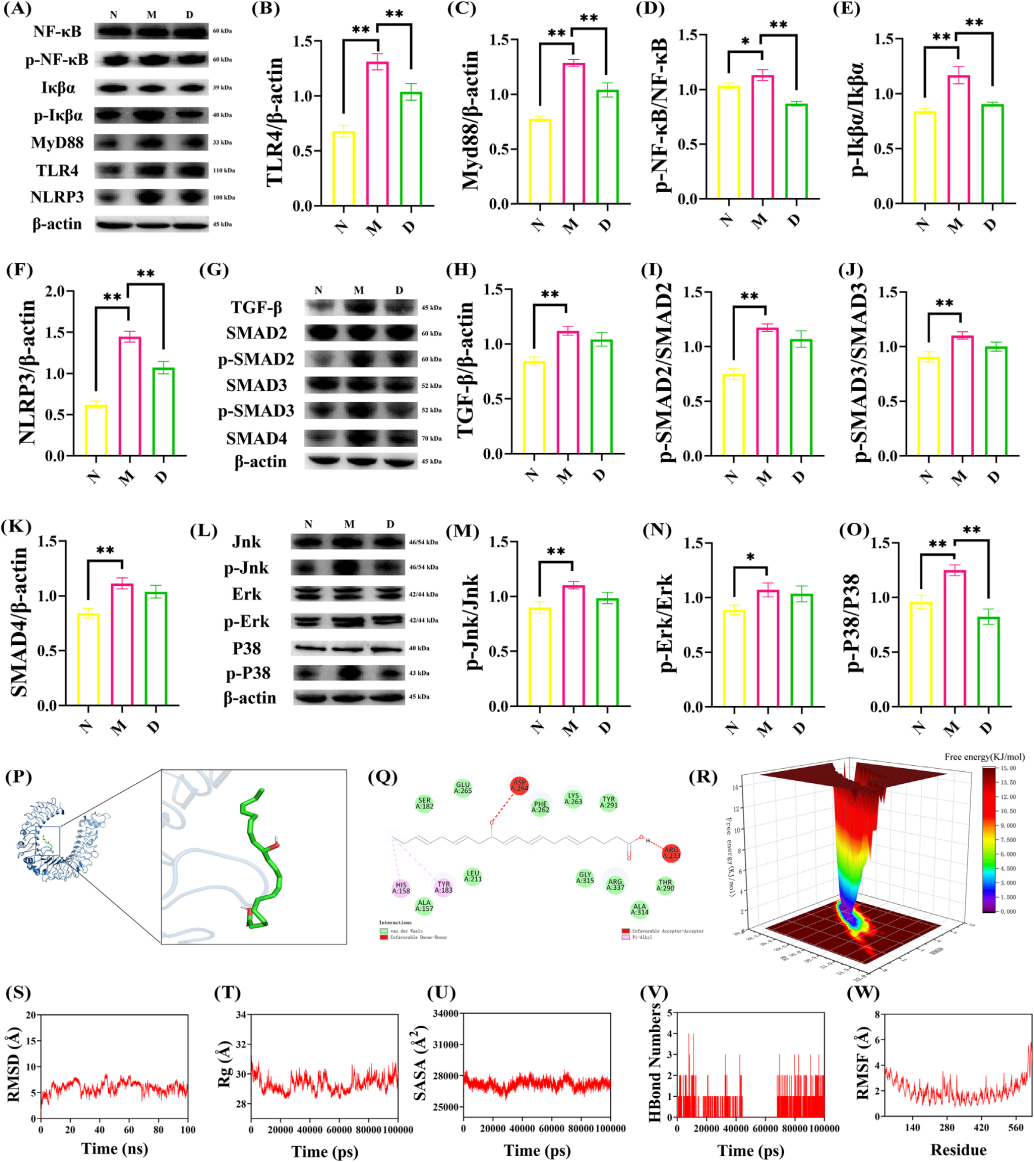
TLR4 as a key receptor at 12‐HEPE exhibited alleviated effect on colitis. (A) The protein levels of NF‐κB, p‐NF‐κB, IκBα, p‐IκBα, MyD88, TLR4 and NLRP3; (B‐F) The relative intensities of TLR4/β‐actin (B), MyD88/β‐actin (C) p‐NF‐κB / NF‐κB (D) p‐IκBα/ IκBα (E), NLRP3/β‐actin (F); (G) The protein levels of TGF‐β, SMAD2, p‐SMAD2, SMAD3, p‐SMAD3, and SMAD4; (H‐K) The relative intensities of TGF‐β/β‐actin (H), p‐SMAD2/ SMAD2 (I), p‐SMAD3 / SMAD3 (J), SMAD4/β‐actin (K); (L) The protein levels of Jnk, p‐Jnk, Erk, p‐Erk, P38 and p‐P38; (M‐O) The relative intensities of p‐Jnk / Jnk (M), p‐Erk / Erk (N) p‐P38 / P38 (O). (P‐Q) Molecular docking of 12‐HEPE with TLR4. (R) Gibbs energy landscape (3D). RMSD (S), Rg (T), SASA (U), H‐bond number (V), and RMSF (W) analysis of 12‐HEPE binding to TLR4.

As mentioned above, SCP treatment and fecal microbiota transplantation of SCP mice can enhance the concentration of 12‐HEPE. Thus, the mice were injected with 12‐HEPE to verify the effect. As expected, mice treated with 12‐HEPE attenuated colitis symptoms, similar to SCP and fecal microbiota transplantation of SCP mice treatment, including body weight, length of colon, fecal water content, fecal pH, and DAI index (Figure 7B–H). Besides, 12‐HEPE can attenuate inflammatory infiltration and disruption of intestinal structure in the colon (Figure 7I). In addition, immunoregulatory effects were measured by observing the change in goblet cells, T cells, and macrophages, which also proved that 12‐HEPE could significantly enhance the goblet cells and reduce the number of CD4^+^, CD8^+^, and F4/80 cells (Figure S10A–E). Compared with M group, the expression of TNF‐α (Figure S10F), IL‐1β (Figure S10G), IFN‐γ (Figure S10H), MPO (Figure S10N), SIgA (Figure S10O), LBP (Figure S10Q), and CCL2 (Figure S10R) in 12‐HEPE‐treat mice was significantly reduced. And the levels of IL‐2 (Figure S10I), IL‐4 (Figure S10J), IL‐13 (Figure S10K), IL‐17 (Figure S10L), IL‐22 (Figure S10M), and DAO (Figure S10P) in D groups were improved compared with DSS‐induced colitis mice, similar to SCP or SCP‐trans mice, and in contrast with antibiotics treatment mice. For intestinal barrier function, the expression of Claudin‐1 and ZO‐1 was significantly recovered (*p* < *0.01*) in 12‐HEPE‐treated mice compared with the DSS‐induced colitis mice (Figure S10S–V). The results determined by IHC in MUC‐2 protein were verified by the above results of intestinal barrier function (Figure S10W,X). Furthermore, in the signaling pathway, compared with the M group, 12‐HEPE inhibits the phosphorylation of NF‐κB, iκBα, Jnk, Erk, P38, SMAD2, and SMAD3, and the TLR4, Myd88, TGF‐β3, and SMAD4 expression in colitis mice. However, only the related proteins of the TLR4‐mediated NF‐κB signaling pathway, including TLR4 (*p* < *0.01*), Myd88 (*p* < *0.01*), NF‐κB (*p* < *0.01*), and iκBα (*p* < *0.01*) were significantly inhibited (Figure 8A–O). Thus, the protective effects of SCP against DSS‐induced colitis depend on the enrichment of 12‐HEPE mediated by microbiota.

### 2.8 Computational Docking Simulations and MD Analysis of 12‐HEPE Binding to Key Receptor TLR4

To further verify whether 12‐HEPE can interact with the TLR4 receptor, the Discovery Studio molecular docking was used to simulate the interaction of 12‐HEPE with the TLR4 receptor. 12‐HEPE could stably bind to TLR4 with binding free energies of −6.1 kcal/mol (Figure 8P), indicating that the two have good binding activity. Thereinto, the HIS158 and TYR183 residues on the TLR4 receptor formed hydrophobic interactions with 12‐HEPE. The ASP264 and ARG233 residues on the TLR4 receptor formed an Unfavorable Donor‐Donor force with 12‐HEPE. The residues of SER182, GLU265, PHE262, and ALA157 on the TLR4 receptor formed the van der Waals force with 12‐HEPE (Figure 8Q).

Root mean square deviation (RMSD) quantifies the conformational stability of proteins and ligands by calculating the average atomic displacement from a reference structure. Smaller deviations correspond to better conformational stability. Consequently, we employed RMSD to assess the equilibrium state of the simulated system. The complex system reached equilibrium after 80 ns, ultimately fluctuating around 5.2 Å, indicating high stability during small molecule‐target protein binding. Further analysis revealed slight fluctuations in both the radius of gyration (Rg) and solvent‐accessible surface area (SASA) values of the complex system during motion, suggesting that conformational changes occurred in the small molecule‐target protein complex during dynamic movement. Hydrogen bonds are a critical determinant of both binding affinity and specificity in ligand‐protein interactions. During the dynamic simulation, which ranged from 0 to 4, many hydrogen bonds formed between the target protein and the small molecule. Notably, the complex maintained approximately 2 hydrogen bonds for most of the simulation time, demonstrating stable hydrogen bond interactions between the target protein and ligand.

The flexibility of amino acid residues in proteins was reflected by the root mean square fluctuation (RMSF). The RMSF values of the complex system were relatively low (mostly below 4 Å), indicating reduced flexibility and enhanced structural stability (Figure 8R–W). In conclusion, the 12‐HEPE‐TLR4 system is stable in binding, and the complex has a good hydrogen bonding effect. Therefore, 12‐HEPE has a good binding effect on the target protein TLR4.

### 2.9 12‐HEPE Exhibited Protective Effect on Colitis by Disrupting TLR4 Activation

**FIGURE 9:**
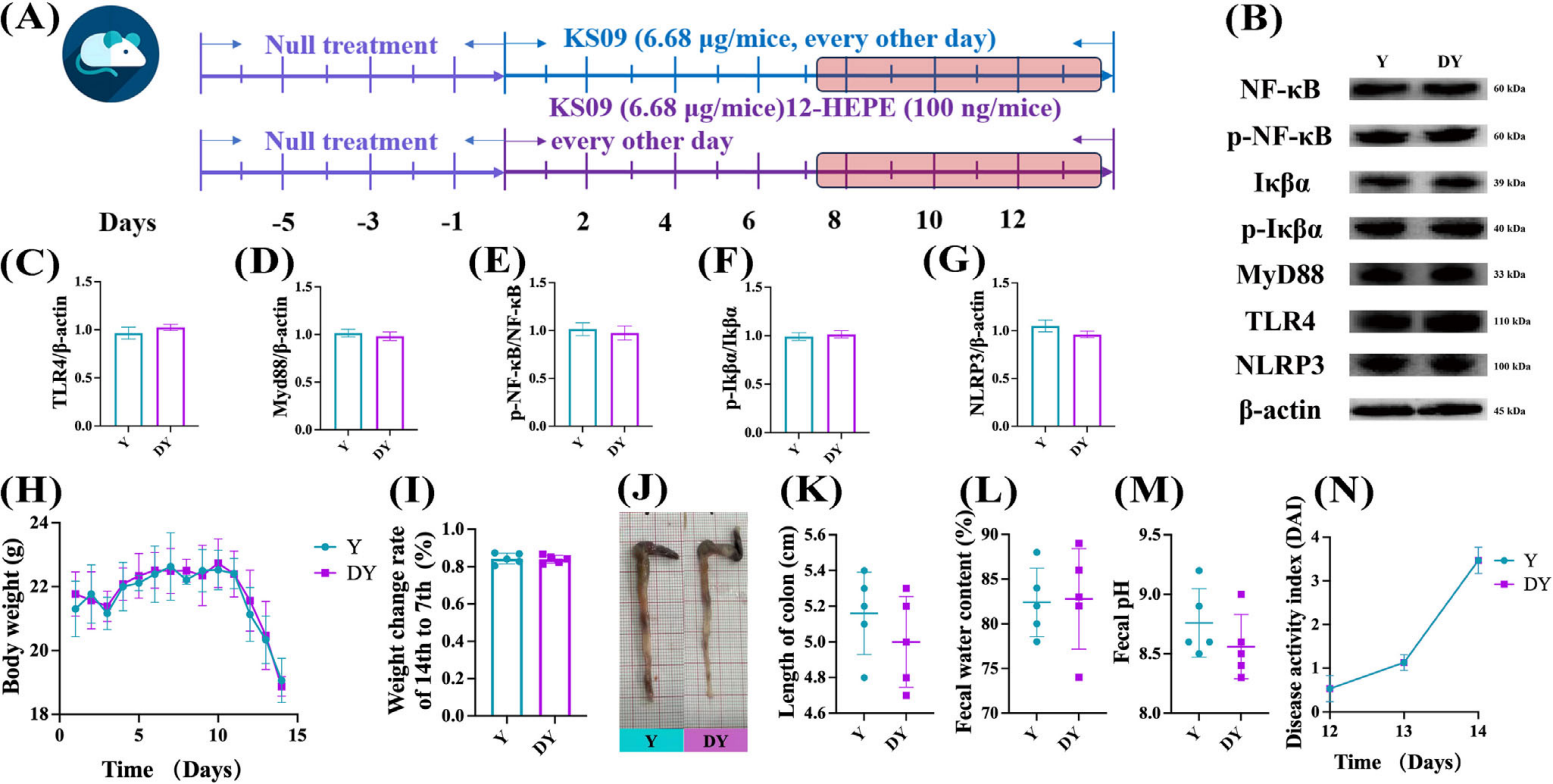
12‐HEPE exhibited a protective effect on colitis by disrupting TLR4 activation. (A) Schematic of the experimental implementation of polysaccharide on DSS‐treated mice; In the study of activator experiment, mice were randomly divided into two groups: KS09 (Y) group and KS09 + 12‐HEPE (DY) group. TLR4 activator KS09 and KS09+ 12‐HEPE had been injected into the N and M groups every other day. Besides, the 3% DSS was added into drinking water during the last 7 days except N group; (B) The protein levels of NF‐κB, p‐NF‐κB, IκBα, p‐IκBα, MyD88, TLR4 and NLRP3; (C–G) The relative intensities of TLR4/β‐actin (C), MyD88/β‐actin (D) p‐NF‐κB / NF‐κB (E) p‐IκBα/ IκBα (F), NLRP3/β‐actin (G); (H) Body weight; (I) Body weight change rate of 14th to seventh; (J) Representative images of the mouse colon; (K) Colon length; (L‐M) Efficacy of TLR4 activator KS09 and KS09+ 12‐HEPE on fecal water content, fecal pH values; (N) DAI; The DAI = (body mass index+ fecal consistency score+ anal bleeding score)/3.

**FIGURE 10:**
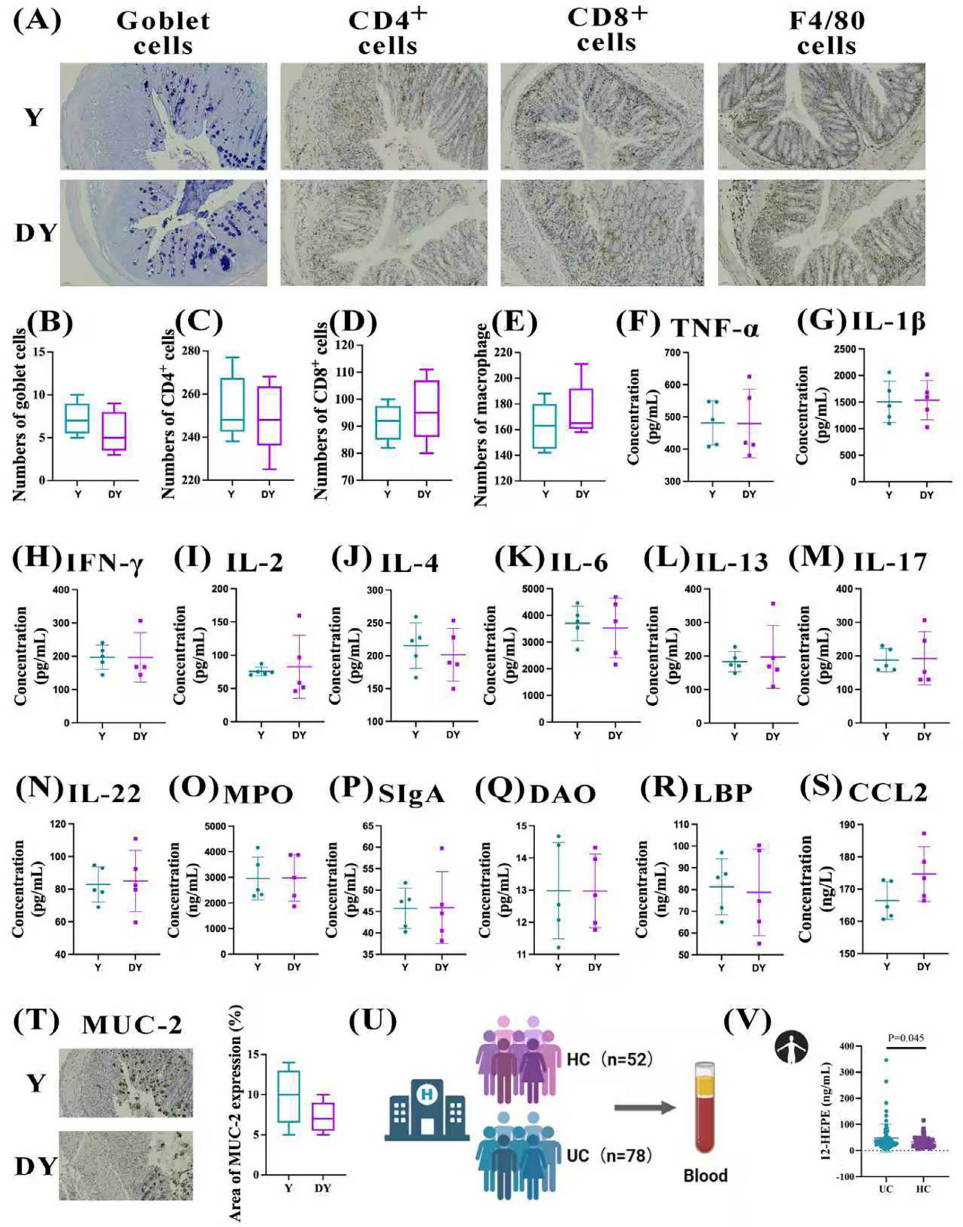
Mechanism of 12‐HEPE exhibited a protective effect on colitis. (A) Immunohistochemical staining of CD4^+^, CD8^+^, F4/80, and goblet cells. Quantitative analysis of assembly diagram with ABPAS (B), CD4^+^ (C), CD8^+^ (D), and F4/80 (E); (F) TNF‐α concentration; (G) IL‐1β concentration; (H) IFN‐γ concentration; (I) IL‐2 concentration; (J) IL‐4 concentration; (K) IL‐6 concentration; (L) IL‐13 concentration; (M) IL‐17 concentration; (N) IL‐22 concentration; (O) MPO concentration; (P) SIgA concentration; (Q) DAO concentration; (R) LBP concentration; (S) CCL2 concentration; (T) Immunohistochemical staining and Quantification of MUC‐2. (U) A discovery cohort and independent validation human UC cohort were established at hospitals; (V) The concentration of 12‐HEPE in HC (*n*  =  52) and UC (*n*  =  78) groups.

TLR4 receptor was an important trigger for colitis progression [29]. The expression of TLR4‐related proteins was elevated in colitis mice. The colitis symptoms could be suppressed by inhibiting TLR4 receptor activation. Thus, TLR4 receptor inhibition in colitis mice is a potential therapeutic target. Encouraged by the result that 12‐HEPE can alleviate colitis symptoms, we intended to figure out whether 12‐HEPE exerts the anti‐inflammatory effect via suppressing TLR4 activation. We used the activator along with 12‐HEPE for intraperitoneal injection to see if 12‐HEPE could still exert a relieving effect on colitis mice. As expected, 12‐HEPE cannot inhibit the phosphorylation of NF‐κB, iκBα, and expression of Myd88, NLRP3 in colitis mice compared with the Y group, which indicates that the TLR4 receptor plays a key role in this process of 12‐HEPE alleviating colitis (Figure 9B–G). In addition, 12‐HEPE can't show any alleviating effect in the mice colitis symptom index, including body weight, the length of colon, fecal water content, fecal pH, and DAI index (Figure 9H–O). Similar results were also observed in immune cell number, cytokine expression, and intestinal barrier integrity, that is, 12‐HEPE can't inhibit TLR4 when KS09 exists (Figure 10A–T). Hence, these results indicated that 12‐HEPE exhibited anti‐inflammatory effects in colitis mice by interrupting the TLR4 receptor.

### 2.10 Elevated Serum 12‐HEPE in UC Patients Suggests a Compensatory Response

To explore the 12‐HEPE with the severity of colitis in humans, the independent validation cohort was enrolled (Figure 10U). Interestingly, we observed that serum 12‐HEPE levels in patients with active colitis were not decreased but significantly higher than those in healthy controls (Figure 10V). This finding presents an intriguing contrast to the therapeutic effect of exogenous 12‐HEPE supplementation in mice. We hypothesize that this elevation does not indicate a pathogenic role but rather represents a compensatory host‐derived protective response to counteract ongoing inflammation. The significant heterogeneity among patients, with a distinct subgroup exhibiting very high 12‐HEPE levels, may reflect varying capacities to mount this protective response.

## 3 Discussion

Polysaccharides linked by glycosidic bonds are a class of natural polymers and are widely found in aqueous extracts of some edible and medicinal plants [30]. *C. paliurus* is a well‐known health‐enhancing herbal tea that contains many bioactive compounds, including polysaccharides, triterpene saponins, and flavonoids. CP is widely regarded as a central active component of *C. paliurus* [31, 32, 33]. However, their performances in food processing and production applications are unsatisfactory because of limitations in their structures and properties [8, 31, 34, 35]. Thus, the structure of CP was modified to SCP, ACCP, and CMCP, and their anti‐inflammation effect in DSS‐induced mice was examined. Among these polysaccharides, SCP showed the best anti‐inflammation effect in DSS‐induced mice with low levels of colitis symptoms, cytokine secretion disorder, intestinal barrier injury, and alteration of gut microbiota (Figures 2, 3, 4, 5; Figures S2–S4). The biological activities of polysaccharides depended on their molecular structure [36]. This enhancing effect may be attributed to the fact that the anionic sulfate groups mimic endogenous glycosaminoglycans (e.g., heparin sulfate), potentially interfering with the docking of pro‐inflammatory ligands (e.g., LPS) to their receptors like TLR4 [8]. Moreover, Sulfated polysaccharides are often more resistant to degradation in the upper GI tract, ensuring greater delivery to the distal gut where they can selectively enrich beneficial bacteria (e.g., SCFA‐producing Bacteroidetes), as clearly observed in our study (Figure 3; Table S5). Further, the structure of SCP was analyzed. The backbone of CP1 consists of 1,3‐Gal and 1,6‐Glc in a ratio of 4:2, with approximately every two circles substituted at the O‐2 and O‐6 positions. The side chains consisted of T‐Araf, T‐Glcp, 1, 4‐Manp, 1, 3‐Araf, and 1, 2, 6‐Manp. Further, the 2D NMR HSQC spectrum showed that at 84.71, 83.82, and 80.11 ppm, these peaks were weakened obviously, which indicated that C4 in residue A, C2 in residue A, and C2 in residue C were primarily substituted by the sulfate group, respectively (Figure 1; Figure S1).

The gut microbiota is instrumental in shaping the development and function of the systemic immune system [37]. It has emerged as a dominant environmental factor in the pathogenesis of colitis [7]. Recently, studies have reported that the polysaccharides have the ability to regulate the ecology of gut microbial [38], and our findings demonstrated that SCP ameliorates disruption of the intestinal tight junction barrier via suppression of the TLR4/NF‐κB pathway. Oral SCP administration improved the antioxidant capacity and immune status of immunosuppressed mice and stimulated the growth of beneficial gut microbiota. However, the role of gut microbiota in the anti‐inflammatory effects of SCP remains unclear. Our experimental results showed that mice exhibited more severe colitis when using antibiotic cocktail therapy, indicating that DSS further exacerbates colitis when the removal of gut microbiota is removed (Figure 4A–I). Further, the protective effect of SCP on joints was diminished when colitis mice were given SCP with antibiotic cocktails. So, the anti‐inflammatory effect of SCP was mediated through gut microbiota (Figure 4A–I). A fecal microbiota transplantation experiment was performed to validate our results, and found that the fecal microbiota of SCP could mimic the anti‐inflammation effect of SCP treatment. Thus, we hypothesized that the anti‐inflammatory benefits of SCP were mediated by gut microbiota (Figure 6A–I).

Marked changes in the gut microbiota were found in the SCP‐treated mice. 16S rRNA sequencing revealed that *Bacteroidetes*, *Eubacterium*, and *Prevotella* were significantly enriched in SCP and SCP fecal microbiota transplantation treatment mice (Figure 5E–J). The gut microbiota modulates host health by generating key compounds, such as SCFAs, LPS, arachidonic acid, timnodonic acid, propionate imidazole, and so on [39]. Eicosanoids are potent regulators of homeostasis and inflammation. They are produced by nearly all human cells, with particularly high levels in inflammatory cells, and are primarily synthesized via the cyclooxygenase (COX), lipoxygenase (LOX), and cytochrome P450 enzymatic pathways, whereas a minor fraction arises from non‐enzymatic free radical mechanisms. The COX pathway generates prostaglandins (PGs) and thromboxanes (TXs); the LOX pathway yields leukotrienes (LTs), lipoxins (LXs), and hydroxyeicosatetraenoic acids (HETEs); and the cytochrome P450 pathway produces epoxyeicosatrienoic acids (EETs) [40]. Notably, the pharmacological inhibition of these enzymes accounts for the mechanism of action of multiple anti‐inflammatory drugs in clinical use. Hence, targeted UPLC‐MS metabolomics using mouse serum and liver samples was performed to identify key metabolites that are responsible for the anti‐inflammation benefits of SCP‐regulated microbiota. 64 related metabolites were determined. Among them, the levels of the host‐derived bioactive lipid mediator 12‐HEPE were significantly elevated in SCP‐treated mice and in mice that received fecal microbiota transplantation from SCP‐treated donors. Conversely, depletion of the gut microbiota via antibiotic cocktail treatment drastically reduced 12‐HEPE levels (Figure S9A–L). These results indicated that the gut microbiota remodeled by SCP was instrumental in promoting the host's production or regulation of 12‐HEPE, positioning it as a key microbiota‐dependent mediator. 12‐HEPE is primarily a host‐derived specialized pro‐resolving mediator synthesized from EPA via 12‐LOX in immune cells (e.g., macrophages, neutrophils) and epithelial cells. It inhibits the inflammation associated with contact hypersensitivity by inhibiting neutrophil infiltration into the skin [41]. Takahiro et al. found that 12‐HEPE inhibited foam cell formation and ameliorated the pathology of atherosclerosis in mice [42]. In addition, *Bacteroidetes* had significantly positive correlations with 12‐HEPE both in liver and serum (Figure S9F). These results revealed that SCP‐rich *Bacteroidetes*, SCFAs, and/or other microbial signals may prime or enhance the capacity of host immune/ epithelial cells to produce 12‐HEPE. However, the precise cellular source and the exact microbial‐derived signals that trigger 12‐HEPE upregulation require further investigation using in vitro co‐culture systems.

TLR4 receptor activation is an important trigger for colitis progression [43]. Compared with those in healthy controls, the expression of TLR4, MyD88, p‐NFκB/ NFκB, p‐iκBα/ iκBα, and NLRP3 in the colitis mice was significantly higher. 12‐HEPE may exert its anti‐inflammatory effect by inhibiting their expression (Figure 8A–F). Thus, the TLR4 receptor activator was used to verify these results. Our experimental results showed that the protective effect of 12‐HEPE on joints disappeared when colitis mice were given a TLR4 receptor activator simultaneously (Figure 9A). Thus, the anti‐inflammatory effect of 12‐HEPE was mediated through the TLR4 receptor. The mechanism by which 12‐HEPE exhibited protective effects in colitis by interrupting TLR4 receptor activation was initially verified. Our multi‐omics and intervention studies unraveled a previously unrecognized mechanism: SCP does not directly suppress inflammation but engages a specific gut microbial community to amplify a host‐derived protective circuit, culminating in the upregulation of 12‐HEPE. This lipid mediator then directly interrupts TLR4 activation, breaking a key inflammatory loop in UC.

However, in humans, we found that the serum 12‐HEPE level in patients with active colitis was not reduced but was higher than that of the healthy controls (Figure 10V). What is particularly important is that we identified a unique subgroup with a high concentration in the patient population. This finding constituted an intriguing paradox with the results of our previous animal experiments, in which exogenous 12‐HEPE supplementation alleviated colitis in mice (Figure 7A–I). We hypothesize that the elevated 12‐HEPE levels in vivo are not a driving factor of the disease, but rather a compensatory endogenous protective response (The Compensatory Mechanism Hypothesis). Upon sensing inflammatory stimuli, the body may upregulate the biosynthesis of such specific, specialized pro‐resolving mediators (SPMs) to actively limit excessive inflammatory progression and promote tissue repair [44, 45, 46, 47]. The substantial inter‐individual variation (heterogeneity) in 12‐HEPE concentrations among patients may reflect differences in their immunometabolism status. Individuals with extremely high 12‐HEPE levels are likely to have activated a more robust protective feedback mechanism in their immune system. This finding holds potential translational value. Serum 12‐HEPE levels may serve as a biomarker for assessing an individual's anti‐inflammatory capacity or disease prognosis, with patients exhibiting higher concentrations likely having a good prognosis. It also provides insights into the development of “individualized supplementation therapy”: patients with insufficient endogenous 12‐HEPE production may derive the greatest benefit from exogenous supplementation.

## 4 Conclusion

Taken together, this study provides a comprehensive mechanistic understanding of SCP's anti‐colitis effects, establishing a causal link among polysaccharide structure, gut microbiota ecology, and host immunometabolism. Multi‐omics integration revealed that SCP enriches SCFA‐producing Bacteroidetes, thereby driving colonic epithelial production of the specialized pro‐resolving mediator 12‐HEPE. 12‐HEPE binds TLR4 with nanomolar affinity, blocks NFκB/NLRP3 signaling, and phenocopies SCP‐mediated protection. Genetic or pharmacologic TLR4 reactivation abolishes both 12‐HEPE and SCP efficacy. Collectively, our data establish a druggable microbiota–lipid–TLR4 axis and position SCP as a first‐in‐class, microbiota‐directed therapeutic for UC. The discovery of this SCP‐microbiota‐12‐HEPE‐TLR4 axis not only advances our knowledge of host‐microbe crosstalk but also opens new avenues for treating UC by harnessing the body's endogenous protective mechanisms through microbiota modulation.

## 5 Experimental Section

### 5.1 Extraction and Chemical Modification

Polysaccharide was extracted from *C. paliurus* by a previous procedure [32]. Briefly, the *C. paliurus* (600 g) were added to 95% ethanol overnight for removing pigments, small molecules, and other impurities. The sediment was redissolved and heated to 90°C for 3 h. The supernatant was soaked in 80% alcohol overnight. Then, the sediment was redissolved, and proteins were eliminated using the Sevag and papain method. Following dialysis, the solution was freeze‐dried to obtain the polysaccharide (CP).

Sulfated *C. paliurus* polysaccharide (SCP) was prepared by the chlorosulfonic acid‐pyridine method [35]. The CP was mixed in formamide and reacted with esterification reagent (with a ratio of chlorosulfonic acid to pyridine of 1:8) at 60°C. After that, the solution was adjusted to neutrality and dialyzed using distilled water for 48 h. The dialysate was mixed with 95% ethanol overnight and redissolved with distilled water. The SCP was obtained by lyophilization.

Acetylated *C. paliurus* polysaccharide (ACCP) was prepared by a previous method [48]. Acetic anhydride was added to the flask and mixed with the CP solution. NaOH solution was used to maintain the pH at 8.0–8.5. After that, the solution was adjusted to neutrality and dialyzed using distilled water for 48 h. The dialysate was mixed 95% ethanol overnight and redissolved with distilled water. The ACCP was obtained by lyophilization.

Carboxymethylated *C. paliurus* polysaccharide (CMCP) was prepared by a previous method [33]. The CP was dissolved in 20% NaOH solution and stirred vigorously for 1 h. Then, chloroacetic acid was added to it at 55°C for 5 h. After that, the solution was adjusted to neutrality and dialyzed using distilled water for 48 h. The dialysate was mixed with 95% ethanol and then redissolved. The CMCP was obtained by lyophilization.

The overall total sugar contents of CP, SCP, ACCP, and CMCP were 60.62% ± 0.88%, 49.71% ± 0.56%, 64.89% ± 0.76%, and 55.56% ± 1.13%, respectively. The protein contents of CP, SCP, ACCP, and CMCP were 7.57% ± 0.26%, 6.58% ± 0.13%, 7.25% ± 0.12%, and 6.31% ± 0.29%, respectively. The uronic acid contents of CP, SCP, ACCP, and CMCP were 16.14% ± 0.44%, 21.03% ± 0.35%, 15.78% ± 1.09%, and 16.14% ± 0.44%, respectively [31, 34].

### 5.2 Purification

CP was mixed with 5% H_2_O_2_ at 65°C. After dialysis and freeze‐drying, the decolorized polysaccharide was obtained. Next, 10 mg/mL decolorized polysaccharide was put into a DE‐52 column and eluted using NaCl solution (0.1 m). The eluted solution was collected, dialyzed, and lyophilized, and the pure polysaccharide was obtained, namely CP1. The sulfated polysaccharide was obtained using the same method shown in Section 1, namely SCP.

### 5.3 Structure Analysis

The contents of uronic acid, total sugar, and protein in CP1 and SCP1 were measured by the sulfate‐carbazole method [49], phenol‐sulphuric acid method [50], and Coomassie brilliant blue method [51].

The monosaccharide constituents of CP1 and SCP1 were measured using high‐performance anion‐exchange chromatography via H_2_SO_4_ hydrolysis. The polysaccharide samples were disassembled by 12 m H_2_SO_4_ at 105°C and then detected by HPAEC‐PAD (Dionex ICS‐5000, Thermo Fisher Corp., USA).

A scanning electron microscope (SEM, JSM6701F, Japan) was employed to observe the micromorphologies of CP1 and SCP1 at an accelerating voltage of 5.0 kV.

The polysaccharide was ground with potassium bromide (KBr) and pressed into a homogeneous pellet. The FT‐IR spectrum was then recorded directly from this KBr pellet.

The methylation of CP1 was performed in DMSO with CH_3_I, following activation with NaOH, in an ice bath under dark conditions. The organic phase obtained after CH_2_Cl_2_/water partitioning was dried under a nitrogen stream. The product was then hydrolyzed with TFA, reduced with NaBH_4_, and acetylated with acetic anhydride to yield the PMAAs, which were identified by GC‐MS (Agilent 7890B/7000D, USA).

The CP1 and SCP1 were redissolved in D_2_O three times after each lyophilization. Then, NMR spectroscopy of the polysaccharide was performed on a Bruker AVANCE III HD 400 MHz spectrometer (Bruker, Switzerland). NMR spectra, including ^13^C, ^1^H, ^1^H‐^13^C heteronuclear single‐quantum coherence spectroscopy (HSQC), ^1^H‐^1^H total correlation spectroscopy (TOCSY), ^1^H‐^13^C heteronuclear multiple‐bond spectroscopy (HMBC), ^1^H‐^1^H nuclear overhauser effect spectroscopy (NOESY), and ^1^H‐^1^H correlation spectroscopy (COSY) were detected at 298 K.

### 5.4 Human Subjects

Participants were enrolled from Xijing Hospital between January 1, 2022, and December 1, 2022, and all provided informed consent. For cohort 1, the 78 UC patients (aged 19–79) were enrolled. Key exclusion criteria comprised a history of other gastrointestinal diseases, malignancies, autoimmune or infectious disorders, severe renal impairment (creatinine >3.0 mg/dL), gastrointestinal surgery within the previous year, or prolonged antibiotic use (>3 days) in the three months prior to enrollment. For cohort 2, there were 52 healthy controls (HC). The inclusion and exclusion criteria were the same to cohort 1. Ethical approval for this study was obtained from the Ethics Committee of Xijing Hospital (KY20212027‐C‐1).

### 5.5 Animal Experiment

All animal experiments were conducted in accordance with the National Research Council's Guide for the Care and Use of Laboratory Animals and were approved by the Animal Care and Use Committee of Nanchang University [SYXK (Gan) 2021‐0004].

C57BL/6J male mice aged 6 weeks were purchased from Vital River Laboratory Animal Technology Co., Ltd. After feeding for 7 days, mice were divided into the Normal (N) group, the Model (M) group, the 5‐aminosalicylic acid (5‐ASA) group (PC group), the CP group, the SCP group, the ACCP group, and the CMCP group. Saline was gavaged into N and M groups from 8th to 21st. And CP, SCP, ACCP, and CMCP with a concentration of 50 mg/kg bw were gavaged from the 8th to 21st day into the CP group, SCP group, ACCP group, and CMCP group, respectively. At the same time, 5‐ASA of 100 mg/kg·bw was used for the PC group. Besides, the 3% DSS was added to the drinking water during the last 7 days, except for the N group (Figure 1A). During the last 3 days, the fecal samples were collected. On the last day, mice were sacrificed, and their liver, cecum, colon, and spleen were gathered for further assays. The ingredients of the antibiotic cocktail were metronidazole, penicillin, vancomycin, and neomycin, with dosages of 1000, 1000, 500, and 1000 µg/mL, respectively.

In the study of fecal microbiome removal, mice were randomly divided into the Antibiotics (A) group, Normal (N) group, Model (M) group, and Antibiotics + SCP (AS) group. From the 8th to the 21st, the N and M groups were gavaged with saline, and the A group and AS group were given 0.2 mL of antibiotics and 0.1 mL of antibiotics +0.1 mL 50 mg/kg SCP solution, respectively. During the last 7 days, the drinking water was mixed with DSS (3%) except for the N group (Figure 4A). For the last 3 days, the fecal samples were collected. Finally, mice were sacrificed, and their liver, cecum, colon, and spleen were gathered for further assays.

In the study of fecal microbiome transplantation, mice were divided into the SCP group, the Normal (N) group, the Model (M) group, the FMT group (fecal microbiome transplantation of SCP mice before DSS treatment), and the FMT + SMP group. From the 8th to the 21st, the N and M groups were gavaged with saline, and the FMT group and the FMT + SMP group were given 0.2 mL fecal solution and 0.1 mL fecal solution + 0.1 mL 50 mg/kg SCP solution, respectively. During the last 7 days, the drinking water was mixed with DSS (3%) except for the N group (Figure 5A). For the last 3 days, the fecal samples were collected. Finally, mice were sacrificed, and their liver, cecum, colon, and spleen were gathered for further assays.

In the study of the metabolite injection experiment, mice were divided into three groups: Normal (N) group, Model (M) group, and 12‐HEPE metabolite injection (D) group. From the 8th to the 21st, the N and M groups were gavaged with saline, and 0.2 mL 12‐HEPE at a concentration of 100 ng/mL [42] was injected into the D group. During the last 7 days, the drinking water was mixed with DSS (3%) except for the N group (Figure 7A). For the last 3 days, the fecal samples were collected. Finally, mice were sacrificed, and their liver, cecum, colon, and spleen were gathered for further assays.

In the study of the activator experiment, mice were divided into the KS09 (Y) group and the KS09 + 12‐HEPE (DY) group. TLR4 activator KS09 and KS09 + 12‐HEPE were injected into N and M groups every other day. During the last 7 days, the drinking water was mixed with DSS (3%) except for the N group (Figure 8A). For the last 3 days, the fecal samples were collected. Finally, mice were sacrificed, and their liver, cecum, colon, and spleen were gathered for further assays.

### 5.6 Basic Pathological Feature Experiment

The fecal samples were dried until a steady weight, and the change in fecal weight was recorded. Besides, fecal samples were homogenized and further centrifuged to detect the pH using a pH meter.

The disease activity index (DAI) was calculated by body weight change, stool consistency, and bloody stools.

Colon tissue, liver tissue, and spleen tissue were fixed in 4% paraformaldehyde, then mounted, and stained with hematoxylin/eosin (H&E).

### 5.7 Elisa Experiment

Mice colons were homogenized with PBS. After centrifugation, the supernatants were quantified for the concentrations of the immune‐associated protein (TGF‐β3, SIgA, MPO, DAO, CCL2, LBP, and LPS) and cytokines (TNF‐α, IL‐1β, IFN‐γ, IL‐2, IL‐4, IL‐6, IL‐13, IL‐22) by commercial ELISA kits.

### 5.8 Immunohistochemistry

The colon sections from mice were embedded, sectioned, and dehydrated using xylene and ethanol solutions. The antibodies for AB‐PAS, CD4, CD8, F4/80, MUC‐2 were cultivated with samples. Furthermore, the mouse anti‐rat IgG HRP antibodies were used to permeate the samples. Finally, the colon sample was stained and counterstained with the 3,3‐diaminobenzidine substrate chromogen system and hematoxylin

### 5.9 Oxidative Stress Parameters

Liver tissues were homogenized with PBS. Then, the sample was detected by the commercial kits to reflect the concentrations of malonaldehyde (MDA), superoxide dismutase (SOD), total antioxidant capacity (T‐AOC), and catalase (CAT)

### 5.10 RNA‐seq

Mice colon tissues were snap‐frozen in liquid nitrogen. RNA sequencing and library construction, including fragmentation to 200–300 bp, were commercially performed by Shanghai Personal Biotechnology Co., Ltd. The quality of the final libraries was evaluated using an Agilent 2100 Bioanalyzer (Agilent, USA).

### 5.11 Western Blot

The colon tissue was homogenized with the mixture solution (IP lysis buffers and PMSF at a ratio of 99:1). After centrifugation, the loading buffers were added to the supernatant and heated at 95°C. The protein sample was separated by electrophorese at 220 v for 35 min, and the blocking buffer was added to the sample for 1 h. Primary antibody diluted 1000–2000‐fold was cultured with the sample overnight and then treated with secondary antibodies. Finally, SuperECL Plus (Beyotime, Shanghai, China) was used to detect the expression of the protein.

### 5.12 Short Chain Fatty Acids (SCFAs)

The PBS was added to the cecum content and homogenized using a grinder. Following centrifugation at the condition of 10 000 g at 4°C for 10 min, the 50% (v/v) sulfuric acid and anhydrous ethyl ether were used to separate the sample and obtain the SCFAs. At last, the gas chromatography was used to filter and detect the concentration of the SCFAs.

### 5.13 Molecular Docking

The TLR4 (PDB ID: 2z64) receptor structure was retrieved (https://www.rcsb.org/), while the 12‐HEPE ligand structure was acquired from PubChem. The docking grid of TLR4 was centered on the MD2 hydrophobic pocket, which is the canonical binding site for lipid‐based ligands (e.g., LPS) to activate TLR4. Moreover, 12‐HEPE is a lipid mediator structurally analogous. Our aim was to test if 12‐HEPE could act as a competitive inhibitor by occupying this activation site. Protein preparation, including dehydration and phosphate group removal, was conducted using PyMOL. Molecular docking simulations were performed to investigate protein‐ligand interactions. Prior to docking, both structures were prepared by adding hydrogen atoms to the protein and ligand, removing water molecules from the protein, and defining ligand torsional flexibility. The docking grid coordinates were carefully optimized to encompass the binding site. The optimal binding conformation was selected based on a comprehensive scoring analysis of the docking results. Protein‐ligand interaction patterns were subsequently visualized and analyzed through 2D and 3D representations generated using PyMOL and Discovery Studio 2019, with particular focus on key residue interactions.

### 5.14 Molecular Dynamics (MD) Simulation

MD simulations of 100 ns were conducted using GROMACS 2022 software with the CHARMM36 force field for the protein and GAFF2 for the ligand. The system was solvated using the TIP3P water model in a periodic boundary box extending 1.2 nm from the solute. Electrostatic interactions were calculated using the Particle Mesh Ewald (PME) method with a Verlet cutoff scheme, while van der Waals and Coulombic interactions employed a 1.0 nm cutoff distance. Prior to production runs, the system underwent energy minimization followed by two‐step equilibration: first under NVT conditions (100 ps, 310 K, 0.1 ps coupling time) and then under NPT conditions (100 ps, 1 bar, 0.1 ps coupling time). The final production simulation was performed for 100 ns under NPT conditions at 310 K and 1 bar pressure using a 2 fs timestep.

### 5.15 16S rRNA Gene Sequencing

Following fecal sample collection from mice in sterile isolation cages, microbial DNA was extracted with the E.Z.N.A. Stool DNA Kit. The V3‐V4 regions of the 16S rRNA gene were amplified with primers 338F/806R and sequenced on an Illumina NovaSeq platform (PE250). Subsequent bioinformatic analysis involved OTU clustering with Usearch (v7.0) and diversity analysis with QIIME2 (v1.9.1). Specifically, β‐diversity was visualized through PCoA using the Bray–Curtis distance metric.

### 5.16 Targeted Metabolomics

A total of 100 mg of liver tissue sample (or 100 uL of serum) was taken in a grinding tube, and 40 µL of internal standard (100 ng/mL, eicosanoids) was added. 400 µL of ice methanol (containing BHT and acetic acid) was added, and the samples were left at −80°C, then homogenized twice in a homogenizer and centrifuge (10 000 rpm, 10 min), then the supernatant was removed from the sample and placed in an EP tube (store promptly at −20°C). The supernatant was vortexed for 1 min, centrifuged according to the previous conditions, and the combined supernatant was stored at −20°C. 1.5 mL of Watson water was added to dilute the supernatant.

Then use SPE solid phase extraction: (1) Wash the SPE column with ethyl acetate (3 mL) and methanol (6 mL). (2) Equilibrate with 6 mL of wash solution (0.1% aqueous acetic acid and 5% methanol), do not allow the column to dry out. (3) Load the sample onto the equilibrated column and allow it to settle for a few minutes with the valve closed. (4) Wash with 6 mL of wash solution. (5) Dry the column with a pump under high vacuum for 2 h. (6) Add 10 µL of trapping solution (30% glycerol). (7) 2 mL Elute with methanol and ethyl acetate at a ratio of 1:3 (use low vacuum as needed to get the last droplets out). (8) Nitrogen blow‐dry, re‐dissolve in 200 µL of ice methanol, and transfer to the injection bottle with the cannula.

### 5.17 Data Analysis

All experimental data were expressed as mean ± SD. Duncan's test was performed using IBM SPSS Statistics 26 statistical software. *p* < *0.05* indicated a statistically significant difference, and *p* < *0.01* indicated an extremely significant difference.

## Author Contributions

**Xianxiang Chen**: Writing – original draft, methodology, conceptualization, visualization, and data curation; **Mingyue Shen**: Writing – original draft, methodology, conceptualization, visualization, and data curation; **Rui Zhang**: Writing – review and editing, resources; **Zhibing Huang**, **Hui Niu**, **Qiang Yu**, **Yi Chen**, **Xiangwen Pan**, **Liyuan Rong**, **Huiliang Wen**, **and Jun Yang**: Writing – review and editing; **Jianhua Xie**: Writing – original draft, resources, conceptualization, validation, and supervision.

## Funding

This work was supported by the National Key Research and Development Program of China (2023YFF1104001) and National Natural Science Foundation of China (82460642).

## Conflicts of Interest

The authors declare no conflicts of interest.

## Supporting information




**Supporting File**: advs75681‐sup‐0001‐SuppMat.docx.

## Data Availability

The data that support the findings of this study are available from the corresponding author upon reasonable request.
