# Supplementary material for: Sulfated Cyclocarya Paliurus Polysaccharide Sorchestrates the Gut Microbiome to Mobilize a Host‐Derived 12‐HEPE Against Ulcerative Colitis
Source: Adv Sci (Weinh). 2026 May 15:e75681. Online ahead of print. doi: 10.1002/advs.75681 (PMC13336020; doi:10.1002/advs.75681)
Supplement: Supplementary file 1 — Supporting File: advs75681‐sup‐0001‐SuppMat.docx. [file ADVS-9999-e75681-s001.docx]

**Sulfated *Cyclocarya paliurus* polysaccharide sorchestrates the gut microbiome to mobilize a host-derived army of 12-HEPE against ulcerative colitis**

*Xianxiang Chen^1,4^, Mingyue Shen^1^ ^*^, Rui Zhang^2^, Zhibing Huang^1^, Hui Niu^3^, Qiang Yu^1^, Yi Chen^1^, Xiangwen Pan^1^, Liyuan Rong^1^, Huiliang Wen^1^, Jun Yang^1,4^, Jianhua Xie^1^ ^*^*

^X. Chen, M. Shen, Z. Huang, Q. Yu, Y. Chen, X. Pan, L. Rong, H. Wen, J. Yang, J. Xie^

^1 State Key Laboratory of Food Science and Resources, Nanchang University, Nanchang, 330047, China^

^E-mail: jhxie@ncu.edu.cn (J. Xie); shenmingyue1107@ncu.edu.cn (M. Shen)^

^R. Zhang^

^2 Department of Clinical Laboratory Medicine, Xijing Hospital, Fourth Military Medical University,^ ^Xi'an, 710032, China^

^H. Niu^

^3 Department of Wood Science, The University of British Columbia, Vancouver V6T 1Z4, Canada^

^X. Chen, J. Yang^

^4 College of Food Science and Engineering, Hainan University, Haikou, 570100, China^

**Funding:** National Key Research and Development Program of China (2023YFF1104001).

**Keywords:** *Cyclocarya paliurus* polysaccharide; Microbiota, modification; TLR4; UC

**Figure captions**

**Figure S1** **Sulfated polysaccharide structure analysis.** (A) FT-IR spectra of CP1 and SCP1; (B) The ^1^H- ^13^C HMBC spectrum of SCP1 recorded at 295 K; (C) Main repeating unit of SCP1; (D) Microstructure images of polysaccharide at 200 × and 500 ×.

**Figure S2 The changes of mRNA expression.** (A) Gene coverage analysis; (B) PCoA analysis; (C) The number of genes differing between comparison groups, and the overlap between comparison groups; (D) The genome circle map shows the distribution of genes and the results of significant differences in genes; (E) The up-regulation and down-regulation of the gene in this study, Fold change > 1.5, *p < 0.05*; (F) The GO enrichment analysis; (G) The KEGG analysis; (H) Changes in the number of genes regarding NF-κB between the polysaccharide group and the M group；(I) Changes in the number of genes regarding MAPK between the polysaccharide group and the M group; (J) Changes in the number of genes regarding ECM between the polysaccharide group and the M group.

**Figure S3** **Efficacy of CP, SCP, ACCP and CMCP on the immune regulation and intestinal barrier function in DSS-induced mice.** (A) Immunohistochemical staining of CD4+, CD8+, F4/80 and goblet cells. Quantitative analysis of assembly diagram with ABPAS (B), CD4+ (C), CD8+ (D), and F4/80 (E); (F) TNF-α concentration; (G) IL-1β concentration; (H) IL-4 concentration; (I) IL-13 concentration; (J) IL-17 concentration; (K) IL-22 concentration; (L) MPO concentration; (M) SIgA concentration; (N) TGF-β3 concentration; (O) DAO concentration; (P) LPS concentration; (Q) LBP concentration; (R) The expression levels of occludin Claudin-1 and ZO-1 by western blot; (S-U) The relative intensities of Claudin-1 (S), occluding (T), and ZO-1 (U). (V-W) Immunohistochemical staining and Quantification of MUC-2.

**Figure S4 Polysaccharide inhibited the activation of MAPK, NF-κB and ECM signaling pathway-related proteins.** (A) The protein levels of Jnk, p-Jnk, Erk, p-Erk, P38 and p-P38; (A-D) The relative intensities of p-Jnk / Jnk (B), p-Erk / Erk (C) p-P38 / P38 (D); (E) The protein levels of NF-κB, p-NF-κB, TGF-β, SMAD2, p-SMAD2, SMAD3, p-SMAD3, SMAD4, and MyD88; (F-K) The relative intensities of TGF-β/β-actin (F), p-SMAD2/ SMAD2 (G), p-SMAD3 / SMAD3, (H) SMAD4/β-actin (I), MyD88/ β-actin (J), p-NF-κB / NF-κB (K).

**Figure S5** **Efficacy of SCP mix with antibiotic cocktail on the immune regulation and intestinal barrier function in DSS-induced mice.** (A) Immunohistochemical staining of CD4+, CD8+ and goblet cells. Quantitative analysis of assembly diagram with ABPAS (B), CD4+ (C), and CD8+ (D); (E) TNF-α concentration; (F) IL-1β concentration; (G) IFN-γ concentration; (H) IL-2 concentration; (I) IL-4 concentration; (J) IL-6 concentration; (K) IL-13 concentration; (L) IL-22 concentration; (M) MPO concentration; (N) SIgA concentration; (O) DAO concentration; (P) LPS concentration; (Q) LBP concentration; (R) CCL2 concentration; (S)The expression levels of occludin and Claudin-1 by western blot; (T-U) The relative intensities of Claudin-1 (T), and occluding (U). (V-W) Immunohistochemical staining and Quantification of MUC-2.

**Figure S6 The change in NF-κB and ECM signaling pathway-related proteins when treated with SCP and mix with antibiotic cocktail.** (A) The protein levels of NF-κB, p-NF-κB, IκBα, p-IκBα, MyD88, and TLR4; (B-E) The relative intensities of TLR4/β-actin (B), MyD88/β-actin (C) p-NF-κB / NF-κB (D) p-IκBα/ IκBα (E); (F) The protein levels of TGF-β, SMAD2, p-SMAD2, SMAD3, p-SMAD3 and SMAD4; (G-J) The relative intensities of TGF-β/β-actin (G), p-SMAD2/ SMAD2 (H), p-SMAD3 / SMAD3, (I) SMAD4/β-actin (J).

**Figure S7** **Efficacy of fecal microbiota transplantation on the immune regulation in DSS-induced mice.** (A) Immunohistochemical staining of CD4+, CD8+ and goblet cells. Quantitative analysis of assembly diagram with ABPAS (B), CD4+ (C), and CD8+ (D); (E) TNF-α concentration; (F) IL-1β concentration; (G) IFN-γ concentration; (H) IL-2 concentration; (I) IL-4 concentration; (J) IL-6 concentration; (K) IL-13 concentration; (L) IL-22 concentration; (M) MPO concentration; (N) SIgA concentration; (O) DAO concentration; (P) LPS concentration; (Q) LBP concentration; (R) CCL2 concentration.

**Figure S8 The change intestinal barrier function, NF-κB and ECM signaling pathway-related proteins when treated with SCP and fecal microbiota transplantation.** (A)The expression levels of occluding, Claudin-1 NF-κB, p-NF-κB, IκBα, p-IκBα, MyD88, and TLR4 by western blot; (B-C) The relative intensities of Claudin-1 (B), and occluding (C). (D-E) Immunohistochemical staining and Quantification of MUC-2; (F-I) The relative intensities of TLR4/β-actin (F), MyD88/β-actin (G) p-NF-κB / NF-κB (H) p-IκBα/ IκBα (I); (J) The protein levels of TGF-β, SMAD2, p-SMAD2, SMAD3, p-SMAD3 and SMAD4; (K-N) The relative intensities of TGF-β/β-actin (K), p-SMAD2/ SMAD2 (L), p-SMAD3 / SMAD3, (M) SMAD4/β-actin (N).

**Figure S9** **Targeted UPLC-MS metabolomics using serum and liver samples of mice.** (A-B) Targeted UPLC-MS metabolomics of mice liver; Difference in eicosanoid levels in liver (A); This metabolomics in mice liver reduced in M group compared with N group, reduced in antibiotic cocktail-treated mice and increased in fecal microbiota transplantation mice and SCP-treatment mice (B); (C-D) Targeted UPLC-MS metabolomics of mice serum; Difference in eicosanoid levels in serum (C); This metabolomics in mice serum reduced in M group compared with N group, reduced in antibiotic cocktail-treated mice and increased in fecal microbiota transplantation mice and SCP-treatment mice (D); (E) The metabolomics differing between liver and serum, and the overlap between liver and serum; (F) 12-HEPE in liver and serum were quantified. (G) Spearman’s correlation analysis; Correlation analyses were performed using Spearman’s rank tests; ^*^*p<0.05*, ^**^*p<0.01*.

**Figure S10** **Efficacy of 12-HEPE on the immune regulation and intestinal barrier function in DSS-induced mice.** (A) Immunohistochemical staining of CD4+, CD8+, F4/80 and goblet cells. Quantitative analysis of assembly diagram with ABPAS (B), CD4+ (C), CD8+ (D), and F4/80 (E); (F) TNF-α concentration; (G) IL-1β concentration; (H) IFN-γ concentration; (I) IL-2 concentration; (J) IL-4 concentration; (K) IL-13 concentration; (L) IL-17 concentration; (M) IL-22 concentration; (N) MPO concentration; (O) SIgA concentration; (P) DAO concentration; (Q) LBP concentration; (R) CCL-2 concentration; (S) The expression levels of occludin Claudin-1 and ZO-1 by western blot; (T-V) The relative intensities of Claudin-1 (T), occluding (U), and ZO-1 (V). (W-X) Immunohistochemical staining and Quantification of MUC-2.

**Figure S1**

**
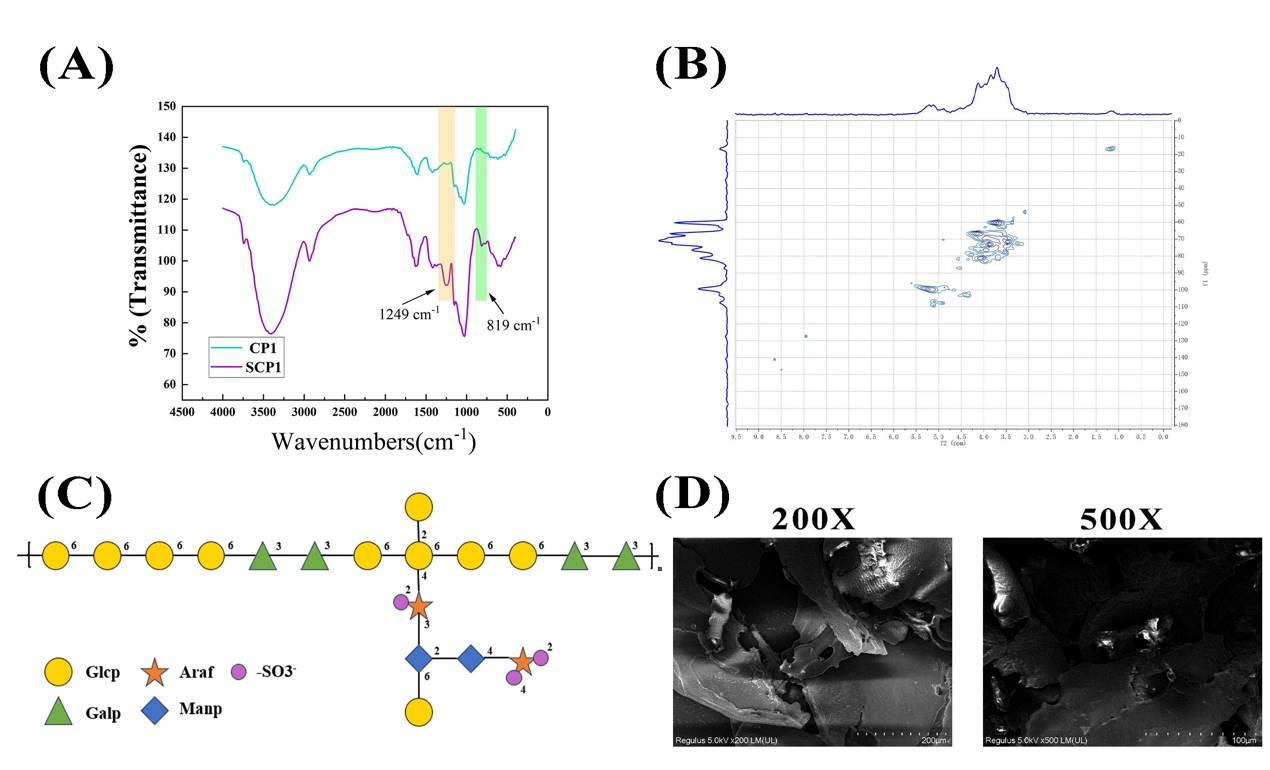
**

**Figure S2**

**
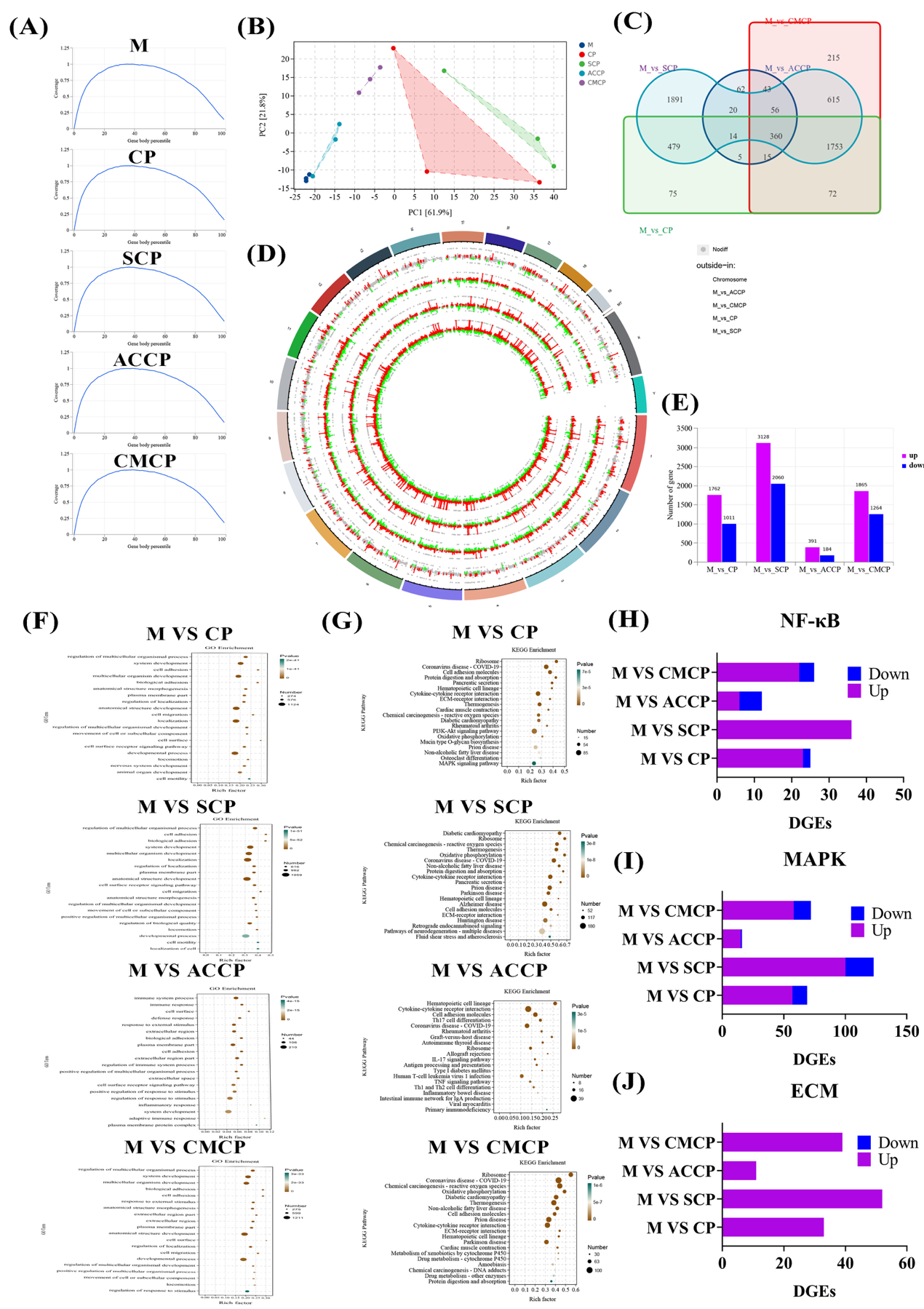
**

**Figure S3**

**
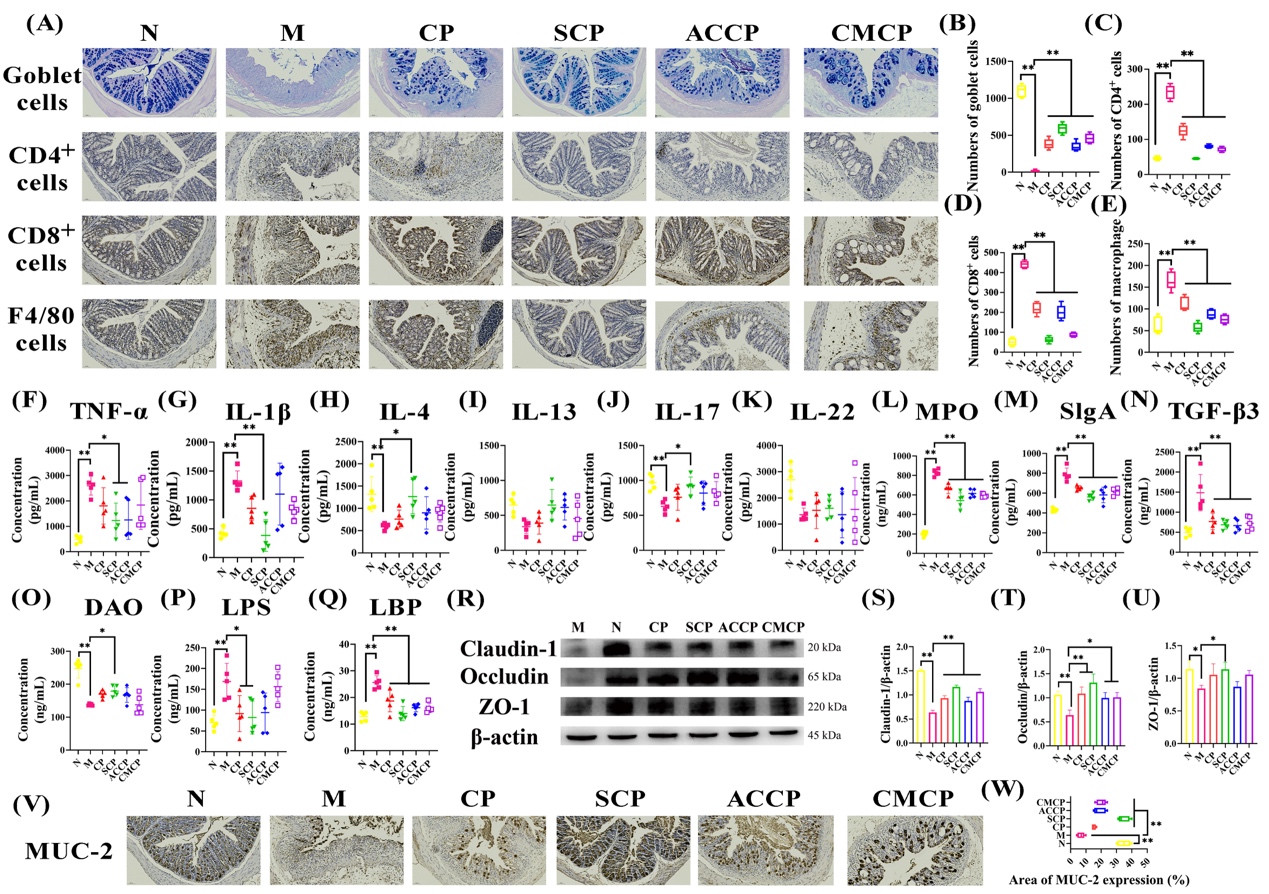
**

**Figure S4**

**
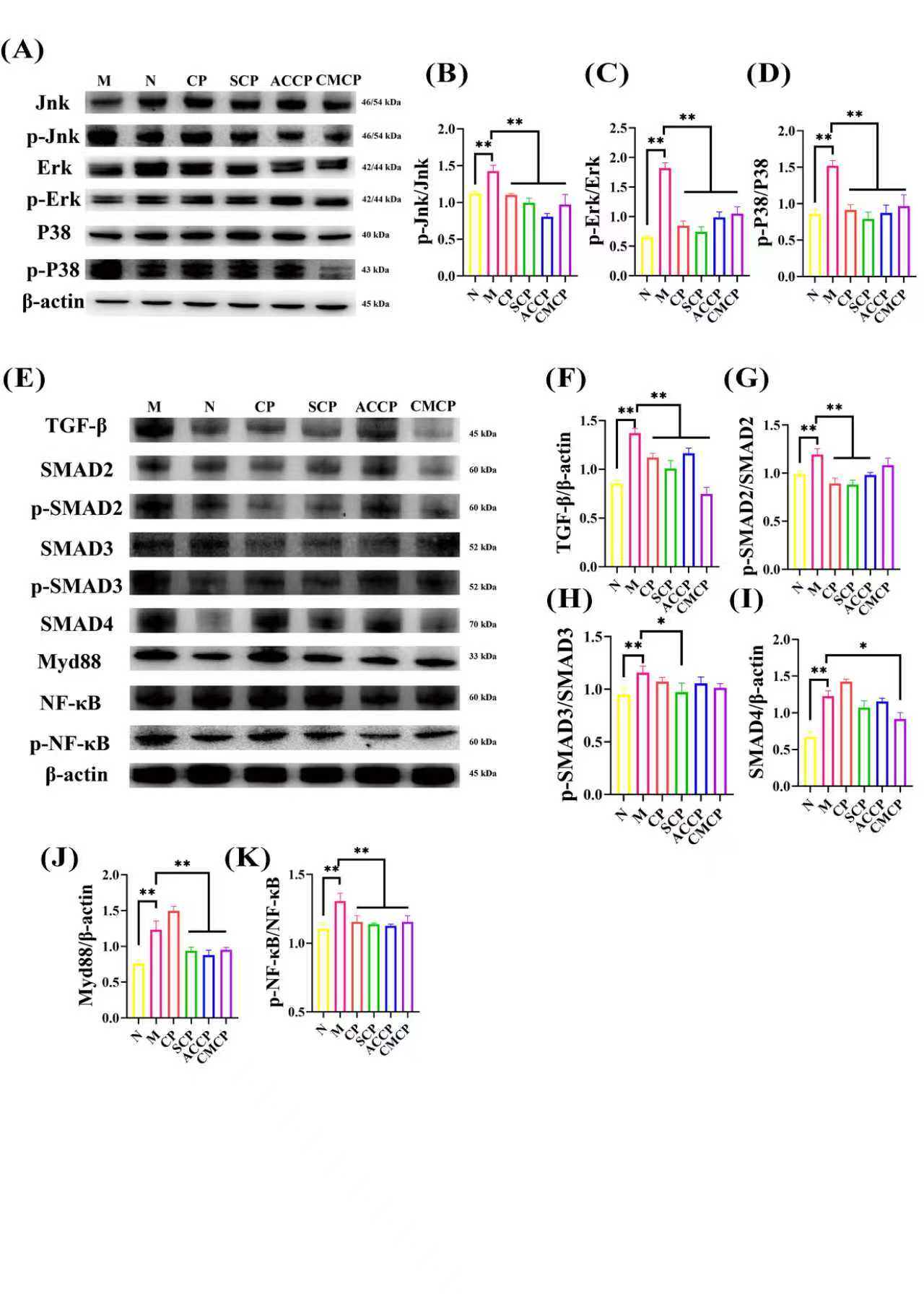
**

**Figure S5**

**
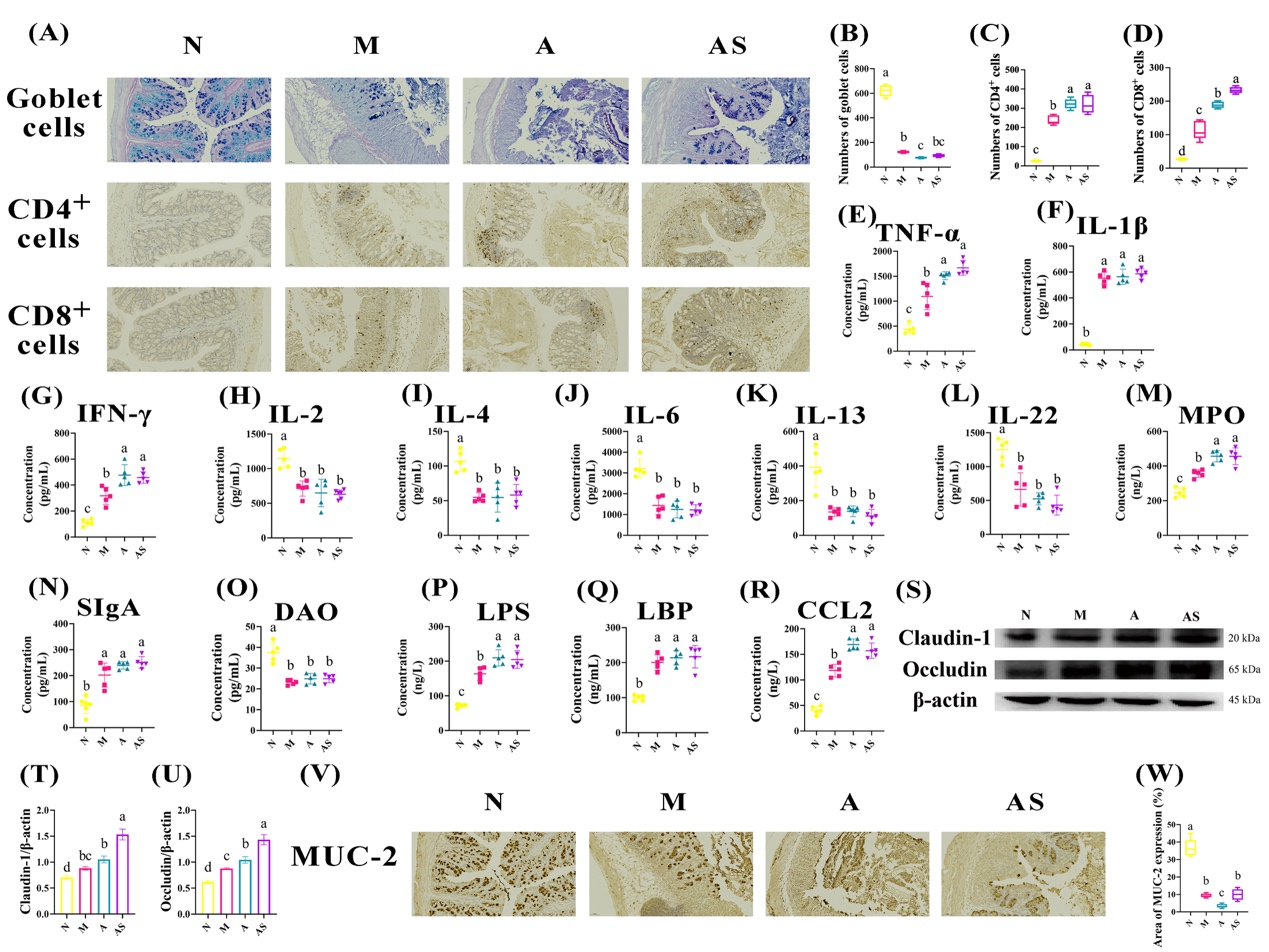
**

**Figure S6**

**
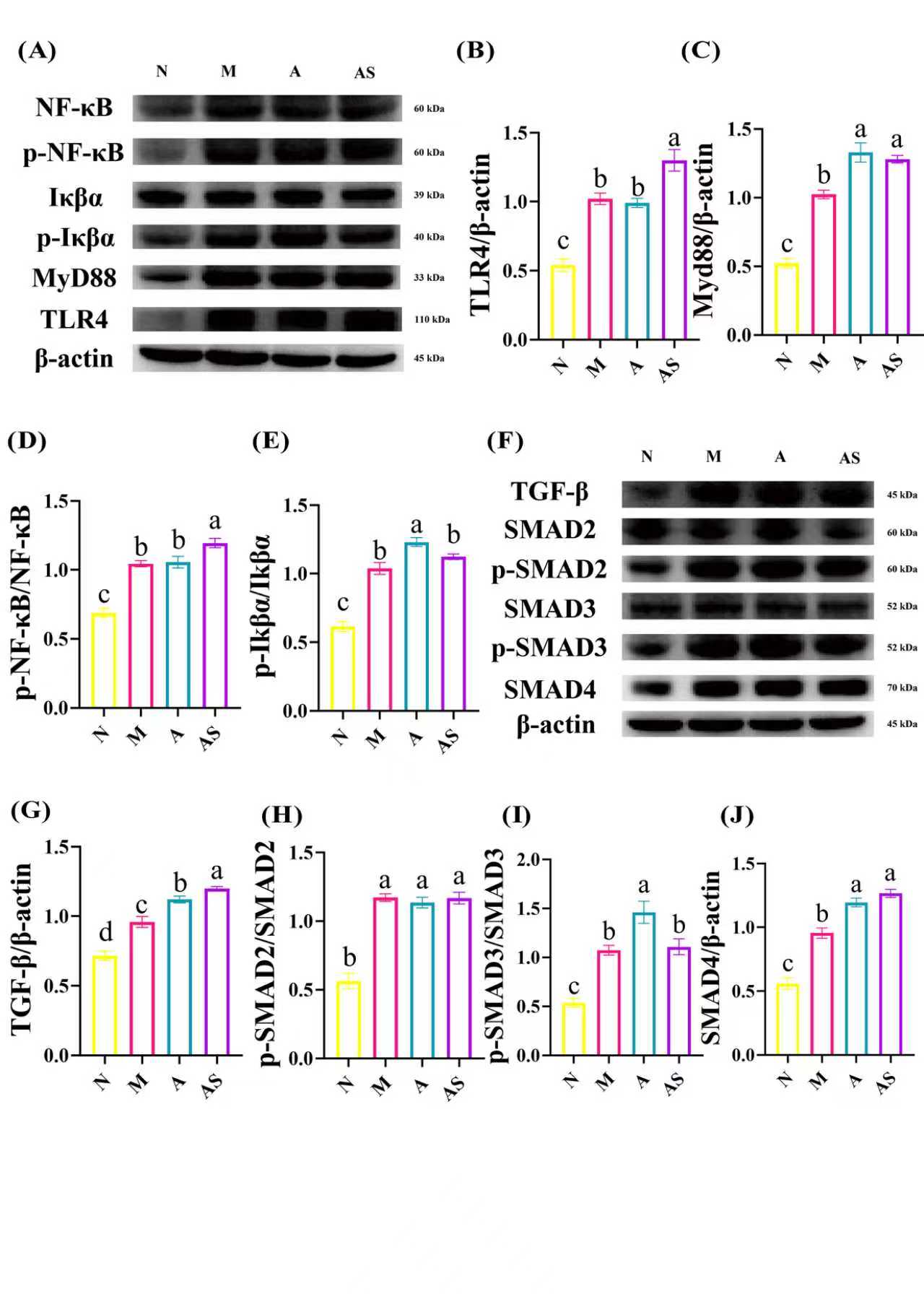
**

**Figure S7**


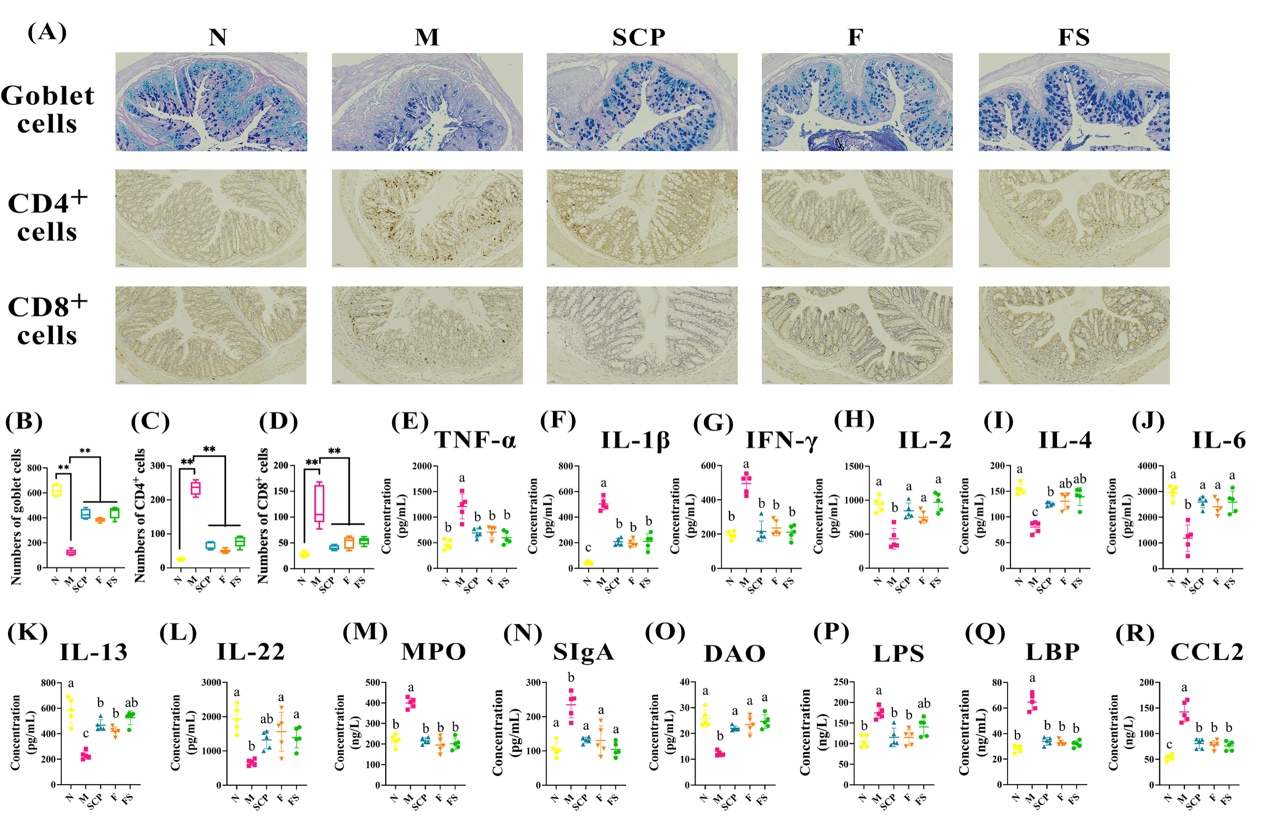


**Figure S8**


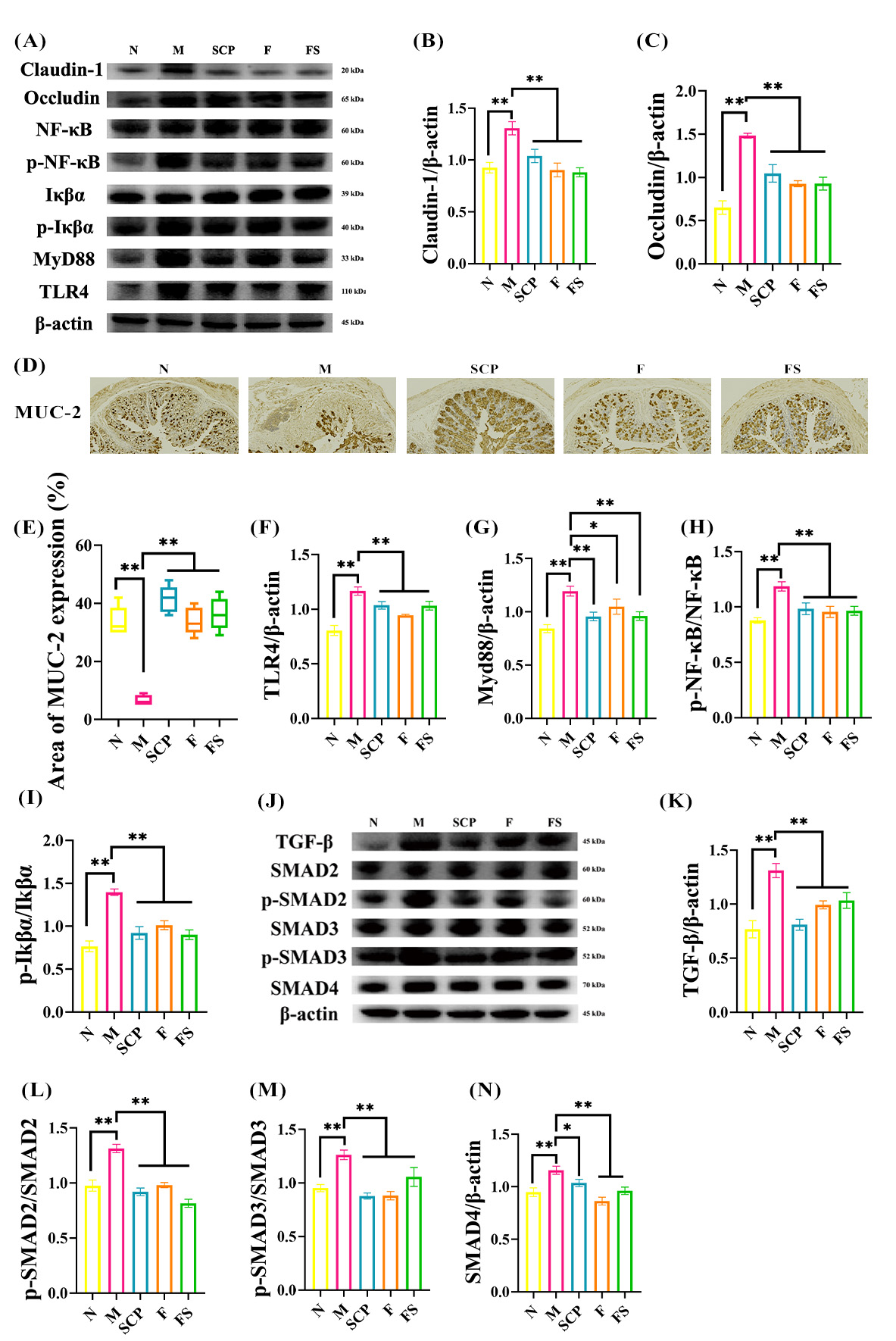


**Figure S9**

**
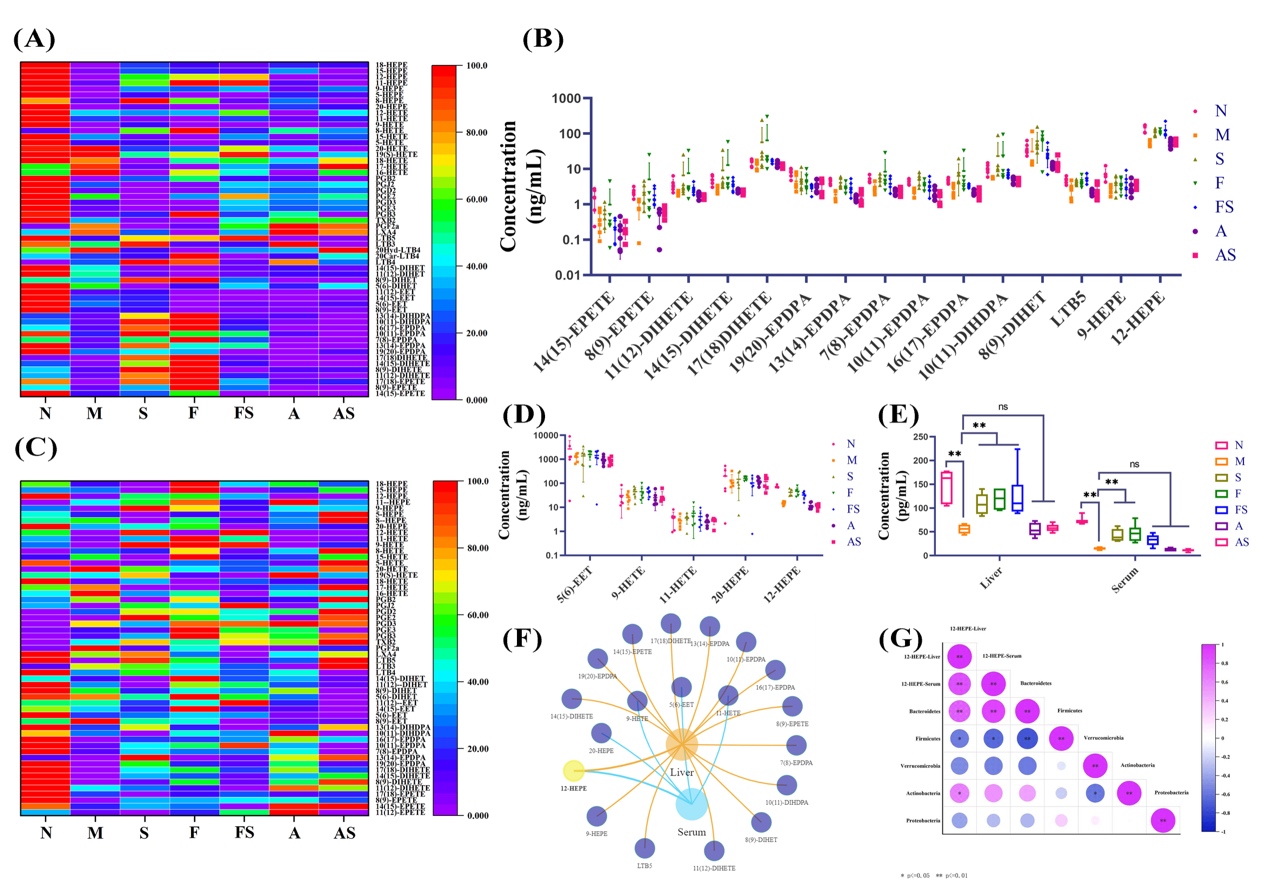
**

**Figure S10**

**
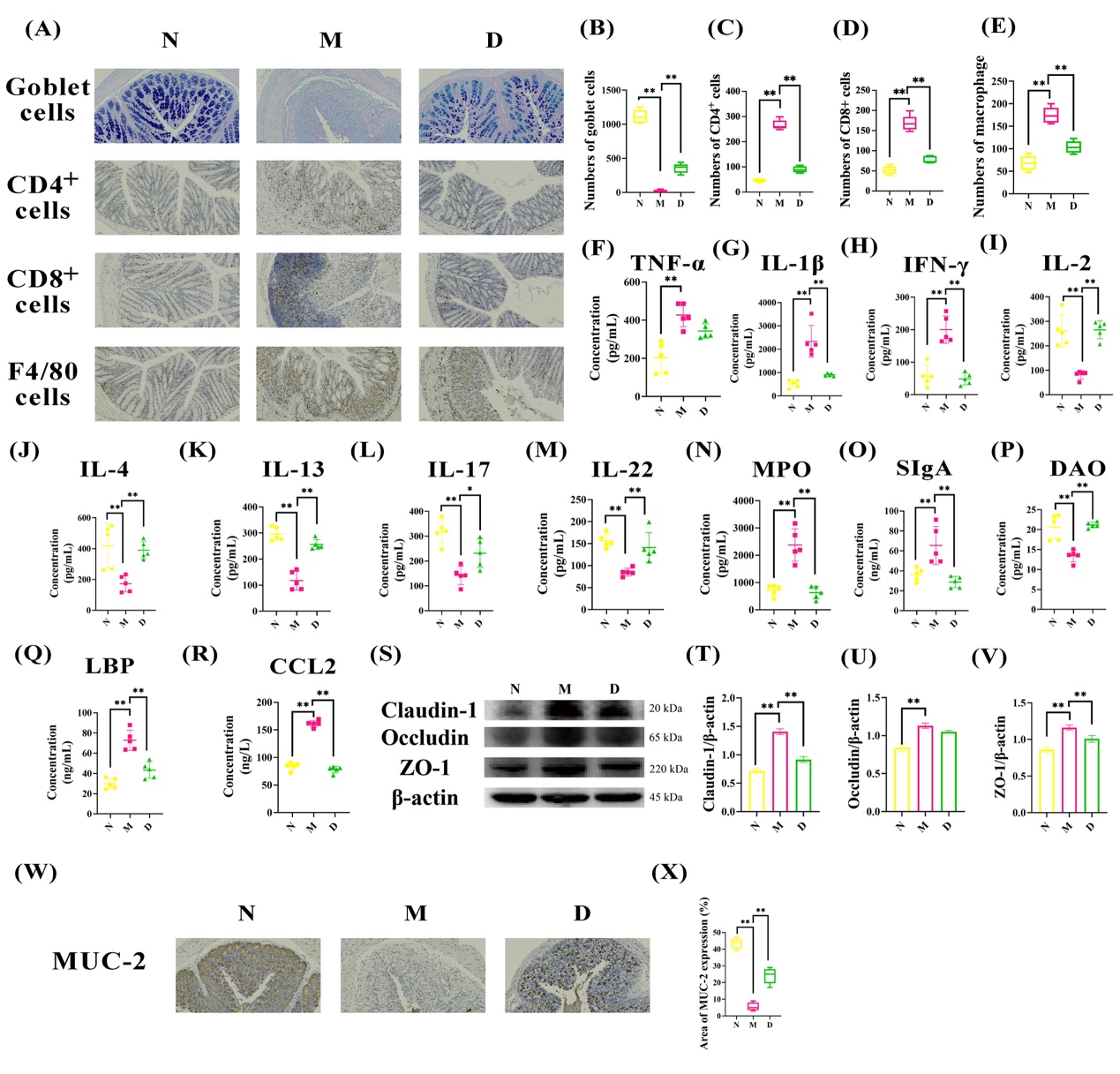
**

**Table captions**

**Table S1** Chemical composition and monosaccharide composition of CP1 and SCP1.

**Table S2** Linkage patterns and corresponding percentages of CP1.

**Table S3** ^1^H and ^13^C NMR chemical shifts of CP1 fraction recorded in D_2_O (ppm).

**Table S4** **Efficacy of CP, SCP, ACCP and CMCP on the level of liver oxidative stress factors in DSS-treated mice**.

**Table S5** **Contents of SCFAs in Cecum**

**Table S6** **Contents of SCFAs in Cecum**

**Table S7** **Contents of SCFAs in Cecum**

**Table S1**

| Samples | CP1 | SCP1 |
| --- | --- | --- |
| Chemical composition (Weight percentage) |  |  |
| Total sugar (%) | 82.14±1.13% | 80.38±1.11% |
| Monosaccharide composition |  | Peak area |
| Ara | 10.23% | 10.22% |
| Gal | 26.70% | 28.14% |
| Glc | 53.57% | 53.99% |
| Man | 9.50% | 8.12% |

Ratio 1, 2 and 3 were calculated based on the peak area of monosaccharides.

**Table S2**

| RT (Min) | PMAA | Linkage pattern | Peak area percentage (%) |
| --- | --- | --- | --- |
| 0.664 | 2, 3, 5-Me_3_-Ara | T-Araf | 7.36% |
| 1.000 | 2, 3, 4, 6-Me_4_-Glc | T-Glcp | 11.43% |
| 1.080 | 2, 4-Me_2_-Ara | 1, 3-Araf | 5.97% |
| 1.356 | 2, 4, 6-Me_3_-Gal | 1, 3-Galp | 24.17% |
| 1.430 | 2, 3, 6-Me_3_-Man | 1, 4-Manp | 4.60% |
| 1.520 | 3, 4-Me_2_-Man | 1, 2, 6-Manp | 3.70% |
| 1.570 | 2, 3, 4-Me_3_-Glc | 1, 6-Glcp | 34.72% |
| 1.942 | 3-Me-Glc | 1, 2, 4, 6-Glcp | 5.07% |

RT: values indicate retention times relative to the PMAA of T-Glcp; Values are calculated according to the ratio of peak areas.

**Table S3**

| Code | Residues | C1/H1 | C2/H2 | C3/H3 | C4/H4 | C5/H5 | C6/H6a/H6b |
| --- | --- | --- | --- | --- | --- | --- | --- |
| A | T-Araf | 107.18  5.11 | 83.82  4.06 | 77.72  3.83 | 84.71  3.94 | -  3.55/3.69 | -  - |
| B | T-Glcp | 99.44  5.31 | 62.80  3.61 | 78.61  3.55 | 72.52  3.35 | 73.79  3.93 | 62.49  3.83/3.76 |
| C | 1, 3-Araf | 107.33  5.15 | 80.11  4.10 | 83.67  4.05 | 82.33  4.26 | 61.66  3.86 |  |
| D | 1, 3-Galp | 98.55  5.26 | 68.96  3.61 | 78.11  3.70 | 70.74  4.07 | 72.01  3.93 | 68.66  3.74 |
| E | 1, 4-Manp | 106.01  4.95 | 70.54  4.03 | 72.12  3.72 | 76.45  3.82 | 74.30  3.58 | 61.33  3.64 |
| F | 1, 2, 6-Manp | 98.09  4.97 | 79.57  4.02 | 71.39  3.73 | 66.61  3.69 | -  3.65 | 67.49  3.84/3.47 |
| G | 1, 6-Glcp | 100.07  5.33 | 73.17  3.55 | 72.22  3.67 | 68.90  3.72 | 74.47  3.70 | 70.01  3.88 |
| H | 1, 2, 4, 6-Glcp | 100.33  4.58 | 72.80  3.19 | 73.33  3.70 | 72.33  3.88 | 76.12  3.78 | 74.58  3.63 |

|  | N | M | CP | SCP | ACCP | CMCP |
| --- | --- | --- | --- | --- | --- | --- |
| T-AOC (mmol/g prot) | 19.88±2.95 | 11.71±3.39^##^ | 17.20±2.05^*^ | 20.54±1.01^**^ | 18.48±1.40^**^ | 17.84±1.58^*^ |
| CAT (Units/mL prot) | 19.16±1.49 | 13.92±0.45^##^ | 15.94±1.65 | 20.01±1.52^**^ | 19.32±1.45^**^ | 16.84±0.86 |
| SOD (U/mg prot) | 99.11±5.68 | 73.21±6.39^##^ | 88.66±3.99^*^ | 96.77±2.68^**^ | 90.75±9.66^**^ | 79.28±5.82 |
| MDA (μmol/mg prot) | 23.72±1.52 | 51.83±1.84^##^ | 44.67±0.85 | 35.65±6.16^**^ | 43.33±5.41^*^ | 44.83±2.22 |

**Table S4**

Values are means ± SD (n=6). *^#^p<0.05* compared with N group, *^##^p<0.01* compared with M group, *^*^p<0.05* compared with M group, *^**^p<0.01* compared with M group.

|  | N | M | CP | SCP | ACCP | CMCP |
| --- | --- | --- | --- | --- | --- | --- |
| Acetic acid | 24.53±6.05 | 7.73±2.86^##^ | 12.98±5.85 | 21.90±2.86^**^ | 21.77±4.20^**^ | 16.58±3.21^*^ |
| Propionic acid | 24.77±2.71 | 4.65±2.65^##^ | 10.68±1.84^**^ | 23.73±6.03^**^ | 17.03±4.38^**^ | 15.36±2.08^**^ |
| Isobutyric acid | 3.22±0.51 | 0.74±0.51^##^ | 1.34±0.38 | 3.21±1.06^**^ | 2.57±0.82^**^ | 1.36±0.44 |
| Butyric acid | 150.11±93.30 | 8.42±4.30^##^ | 65.22±25.46 | 51.50±16.12 | 48.71±20.37 | 32.51±11.33 |
| Isovaleric acid | 5.60±0.51 | 2.86±1.02^##^ | 3.40±0.78 | 5.14±0.81^**^ | 4.16±0.46 | 3.10±0.40 |
| Valeric acid | 7.32±3.13 | 1.59±0.47^##^ | 2.96±1.44 | 6.56±0.67^**^ | 5.04±1.37^*^ | 3.99±1.10 |

**Table S5**

Values are means ± SD (n=6). *^#^p<0.05* compared with N group, *^##^p<0.01* compared with M group, *^*^p<0.05* compared with M group, *^**^p<0.01* compared with M group.

**Table S6**

Values are means ± SD (n=5). *^#^p<0.05* compared with N group, *^##^p<0.01* compared with M group, *^*^p<0.05* compared with M group, *^**^p<0.01* compared with M group.

|  | N | M | A | AS |
| --- | --- | --- | --- | --- |
| Acetic acid | 14.86±0.74 ^a^ | 9.21±2.02 ^b^ | 4.78±1.79 ^c^ | 7.78±2.12 ^b^ |
| Propionic acid | 9.12±0.08 ^a^ | 2.63±1.75 ^b^ | 1.84±0.55 ^b^ | 2.06±0.41 ^b^ |
| Isobutyric acid | 1.31±0.16 ^a^ | 0.70±0.09 ^b^ | 0.46±0.16 ^c^ | 0.66±0.22 ^bc^ |
| Butyric acid | 38.28±8.53 ^a^ | 11.05±5.61 ^b^ | 3.43±1.82 ^c^ | 8.72±2.84 ^bc^ |
| Isovaleric acid | 4.63±0.14 ^a^ | 2.49±0.09 ^b^ | 2.07±0.48 ^c^ | 1.51±0.26 ^d^ |
| Valeric acid | 4.20±0.44 ^a^ | 1.22±0.47 ^b^ | 1.02±0.21 ^b^ | 0.74±0.35 ^b^ |

|  | N | M | S | F | FS |
| --- | --- | --- | --- | --- | --- |
| Acetic acid | 16.09±1.37 ^b^ | 8.02±2.06 ^c^ | 16.84±3.01 ^b^ | 18.46±2.86 ^ab^ | 20.56±1.87 ^a^ |
| Propionic acid | 9.00±0.33 ^b^ | 3.16±1.88 ^c^ | 10.63±1.93 ^b^ | 10.42±1.12 ^b^ | 14.24±2.09 ^a^ |
| Isobutyric acid | 1.40±0.13 ^a^ | 0.38±0.24 ^b^ | 1.33±0.44 ^a^ | 1.39±0.40 ^a^ | 1.48±0.42 ^a^ |
| Butyric acid | 29.29±6.78 ^a^ | 8.31±4.41 ^b^ | 15.61±3.80 ^b^ | 27.15±9.04 ^a^ | 30.52±6.93 ^a^ |
| Isovaleric acid | 4.64±0.25 ^b^ | 1.43±0.44 ^c^ | 4.09±0.84 ^b^ | 5.26±0.91 ^ab^ | 5.96±1.52 ^a^ |
| Valeric acid | 4.12±0.59 ^a^ | 1.02±0.43 ^b^ | 3.81±1.31 ^a^ | 4.46±0.89 ^a^ | 4.96±0.97 ^a^ |

**Table S7**

Values are means ± SD (n=5). *^#^p<0.05* compared with N group, *^##^p<0.01* compared with M group, *^*^p<0.05* compared with M group, *^**^p<0.01* compared with M group.
